# The timing and widespread effects of the largest Holocene volcanic eruption in Antarctica

**DOI:** 10.1038/s41598-018-35460-x

**Published:** 2018-11-22

**Authors:** Dermot Antoniades, Santiago Giralt, Adelina Geyer, Antonio M. Álvarez-Valero, Sergi Pla-Rabes, Ignacio Granados, Emma J. Liu, Manuel Toro, John L. Smellie, Marc Oliva

**Affiliations:** 1 Department of Geography, Centre for Northern Studies & Takuvik Unité Mixte Internationale, Université Laval, G1V 0A6 Quebec, Canada; 2 Institute of Earth Sciences Jaume Almera, ICTJA, CSIC, Lluis Sole i Sabaris s/n, 08028 Barcelona, Spain; 3 Departamento de Geología, Universidad de Salamanca, 37008 Salamanca, Spain; 4 CREAF, 08193 Bellaterra (Cerdanyola del Vallès), Spain; 5 Universitat Autònoma de Barcelona, Bellaterra (Cerdanyola del Vallès), 08193 Spain; 6 Centro de Investigación, Seguimiento y Evaluación, Sierra de Guadarrama National Park, 28740 Rascafría, Spain; 7 Department of Earth Sciences, University of Cambridge, Downing Street, Cambridge, CB2 3EQ UK; 8 Centre for Hydrographic Studies (CEDEX), 28005 Madrid, Spain; 9 Department of Geology, University of Leicester, Leicester, LE1 7RH UK; 10 Department of Geography, Universitat de Barcelona, 08001 Barcelona, Spain

## Abstract

The caldera collapse of Deception Island Volcano, Antarctica, was comparable in scale to some of the largest eruptions on Earth over the last several millennia. Despite its magnitude and potential for far-reaching environmental effects, the age of this event has never been established, with estimates ranging from the late Pleistocene to 3370 years before present. Here we analyse nearby lake sediments in which we identify a singular event produced by Deception Island’s caldera collapse that occurred 3980 ± 125 calibrated years before present. The erupted tephra record the distinct geochemical composition of ejecta from the caldera-forming eruption, whilst an extreme seismic episode is recorded by lake sediments immediately overlying the collapse tephra. The newly constrained caldera collapse is now the largest volcanic eruption confirmed in Antarctica during the Holocene. An examination of palaeorecords reveals evidence in marine and lacustrine sediments for contemporaneous seismicity around the Antarctic Peninsula; synchronous glaciochemical volcanic signatures also record the eruption in ice cores spread around Antarctica, reaching >4600 km from source. The widespread footprint suggests that this eruption would have had significant climatic and ecological effects across a vast area of the south polar region.

## Introduction

Large volcanic eruptions can have pervasive climatic, ecological and economic effects^1^. Climate cooling caused by volcanic aerosols has caused crop failure, starvation, disease and civil unrest and has been linked to the rise and fall of civilisations^2,3^. Although major climate impacts are often associated with tropical volcanoes, high latitude eruptions may also have wide-ranging environmental consequences and trigger pronounced cooling at hemispheric scales^4–6^. Our understanding of the environmental and climatic impacts of many polar volcanic events, however, is limited by a lack of precision regarding the age of past eruptions as well as by the uncertain provenance of numerous tephra layers in ice and sediment cores^7,8^.

**Figure 1:**
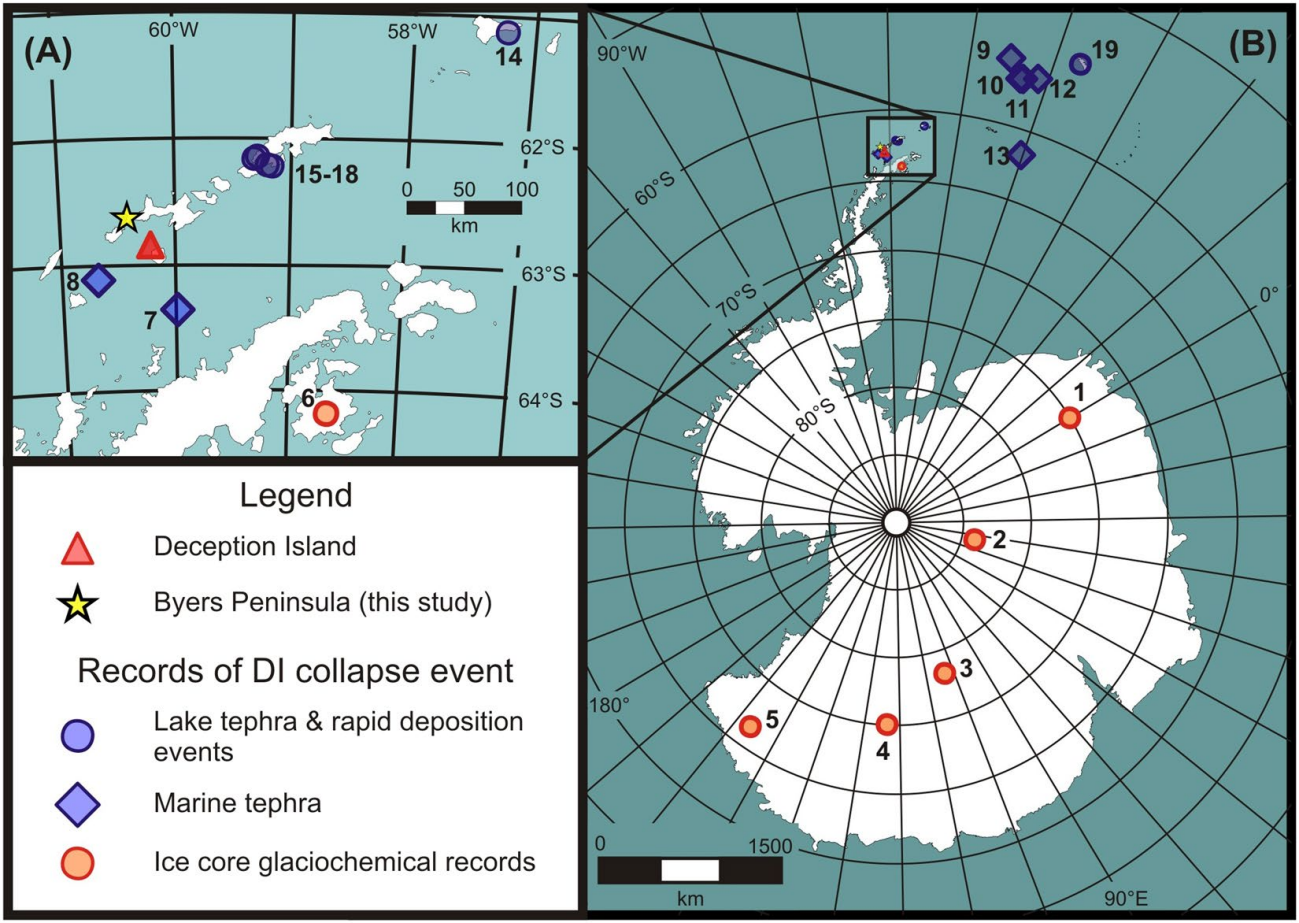
Location map showing (**A**) Deception Island, Byers Peninsula and the Antarctic Peninsula; and (**B**) sites across Antarctica where the Deception Island caldera collapse event is recorded by tephra and/or rapid post-seismic sediment deposition in ice, lake and marine sediment cores. The key for numbered studies can be found in Supplementary Table S3, along with information about how each was linked to the caldera collapse event. Figure 1 was generated with CorelDRAW X3 (www.coreldraw.com) using a Shape file downloaded from the Antarctic Digital Database freely available at http://www.add.scar.org/.

Deception Island is the largest active volcano in the Antarctic Peninsula region; with a basal diameter of 30 km it is on the scale of the volcanoes responsible for the largest eruptions on Earth over the last several millennia^9–12^. It is located in the South Shetland Islands (SSI; Fig. 1), in a volcanically active region that includes nine known volcanoes in the SSI and several more adjacent to the northern Antarctic Peninsula^8^. The potential of Deception Island eruptions to affect climates around Antarctica and beyond is demonstrated by the identification of its tephra layers in marine and lacustrine sediments situated up to ~1300 km from the volcano^8,13–16^ as well as in ice cores from the Antarctic Peninsula and East Antarctica, including at the South Pole^8,17,18^.

**Figure 2:**
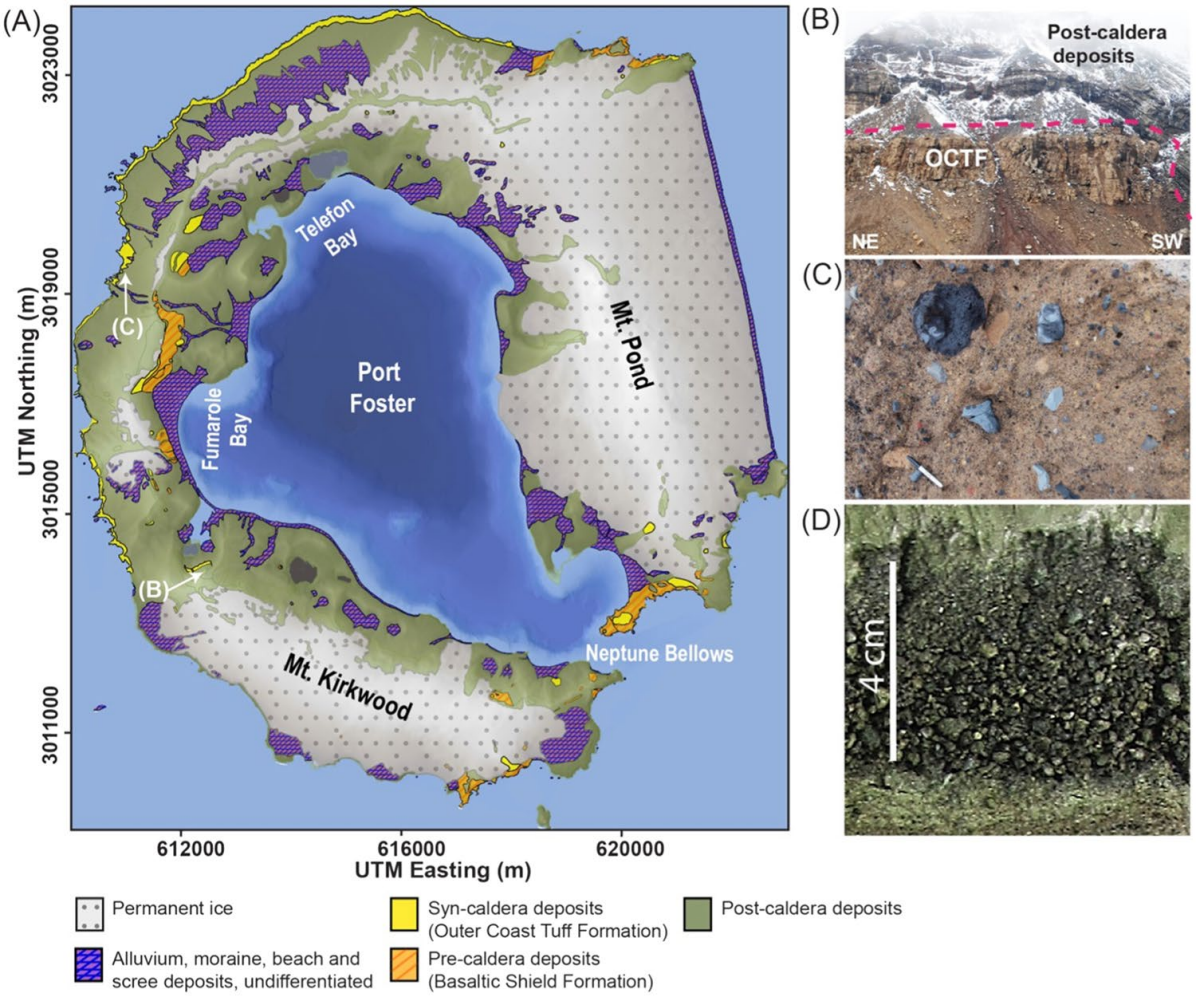
(**A**) Map of Deception Island showing the general geology and outcrop locations of the Outer Coast Tuff Formation (OCTF) (modified from ref.^10^); (**B**) Image of the Vapour Col succession illustrating the contact between the OCTF and post-caldera deposits; (**C**) Detail of OCTF deposits on Deception Island; (**D**) detail of Tephra T3 from Byers Peninsula (Lake Limnopolar, core LIM08_F2B), with alternating organic and mineral sedimentation below T3 and rapid, massive sediment above; note the coarseness of the tephra. (**A**) was generated with QGIS v. 2.18 Las Palmas (available at www.qgis.org) using Shape files and a Digital Elevation Model obtained from the SIMAC geodatabase (described in ref.^52^). Pictures B and C were taken by A.Geyer and published before in ref.^10^. The final layout of this figure was achieved using Adobe Illustrator CC 2015.3.1 (Copyright © 1987–2016 Adobe Systems Incorporated and its licensors).

Deception Island has erupted more than twenty times since the 19^th^ Century including three eruptions between 1967 and 1970 and seismic crises in 1992, 1999 and 2015^10,19,20^. Whilst these recent eruptions have been relatively modest in magnitude, the hitherto undated caldera-forming event ejected a dense-rock equivalent (DRE) of 30–60 km^3^ of magma, comparable in volume to the catastrophic 1815 Tambora eruption that caused global cooling and resulted in “the year without a summer”^1,2,10–12^. The age of this major Deception Island eruption has, however, never been firmly established. Estimates have placed the event between the late Pleistocene and 3370 years before present, with 10000 years ago being the most widely repeated age^12,13,21,22^. During the collapse, rapid volcano-tectonic subsidence along pre-existing, tectonically-influenced faults resulted in a modern 8–10 km-diameter caldera that is of similar dimensions to those of Santorini and Krakatau (refs^10,12^ and references therein; Fig. 2). Caldera collapses on this scale are often associated with intense seismic swarms including multiple high-magnitude earthquakes^23^, and the large volume of magma erupted implies the likelihood of significant, widespread climate impacts.

**Figure 3:**
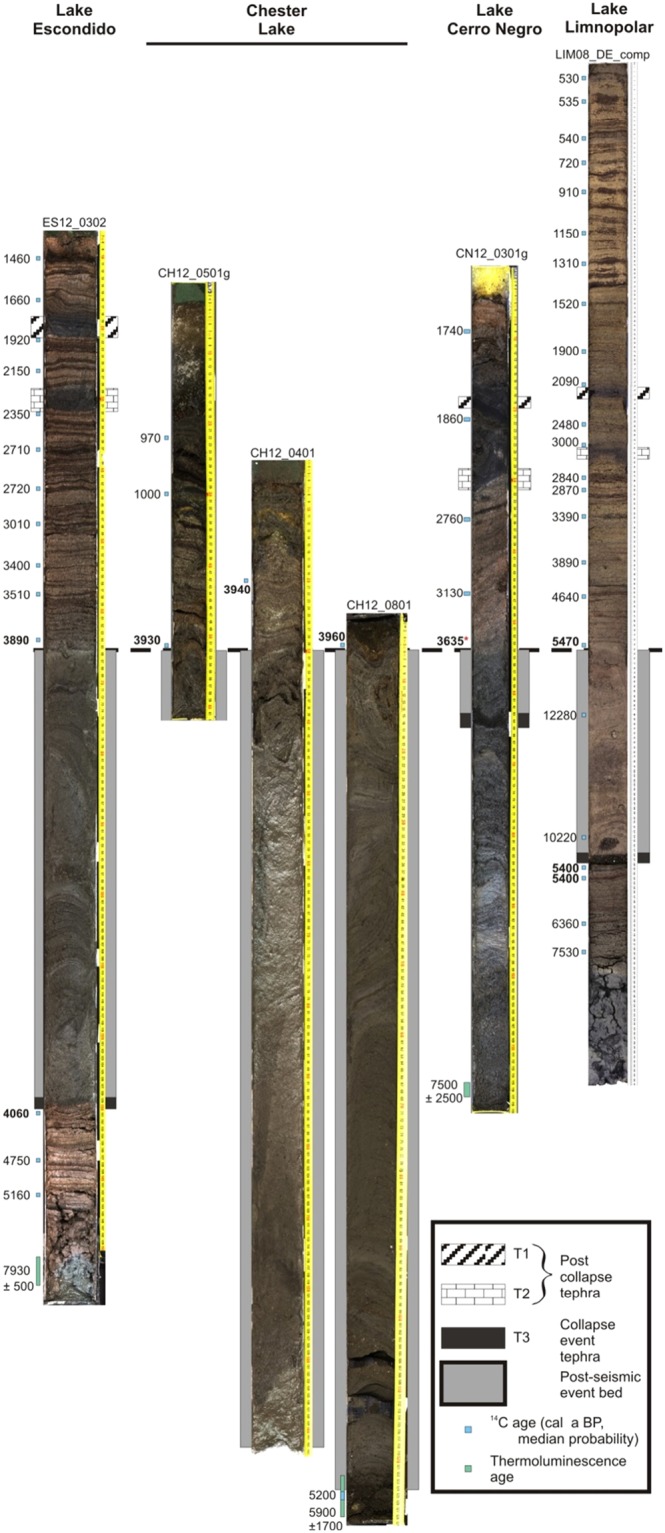
Composite image showing sediment cores from four Byers Peninsula lakes with radiocarbon and thermoluminescence ages (see refs^30,31^ and Supplementary Table 1). Note that the cores are arranged to align the top of the rapid post-seismic depositional unit of varying thickness, interpreted as a mass-wasting deposit, which interrupts the laminated, moss-rich, lacustrine sedimentation in each lake.

Byers Peninsula, located ~40 km northwest of Deception Island on Livingston Island, is ideally located to record the eruptive history of the Deception Island volcano due to its proximity and the presence of numerous lakes. In this study, we examined sediments from Byers Peninsula lakes that preserve detailed, long-term environmental records of Deception Island volcanic activity in the form of tephra deposits. We made direct compositional comparisons between Byers Peninsula tephras and those on Deception Island to provide significantly improved age constraints for the caldera-forming eruption. Here we present data from four Byers Peninsula lakes (Escondido, Cerro Negro, Chester and Limnopolar) in which three major tephra horizons – here referred to as T1, T2, and T3 and whose composition indicated a Deception Island provenance – were correlated based on geochemistry and physical properties^15^ (see Fig. 3 and Supplementary Fig. S1). Our integration of the cumulative evidence derived from geochemical, petrological and palaeolimnological studies provides significant new insights into the chronology and physical and chemical processes that occurred during the Deception Island caldera collapse.

## Results and Discussion

**Figure 4:**
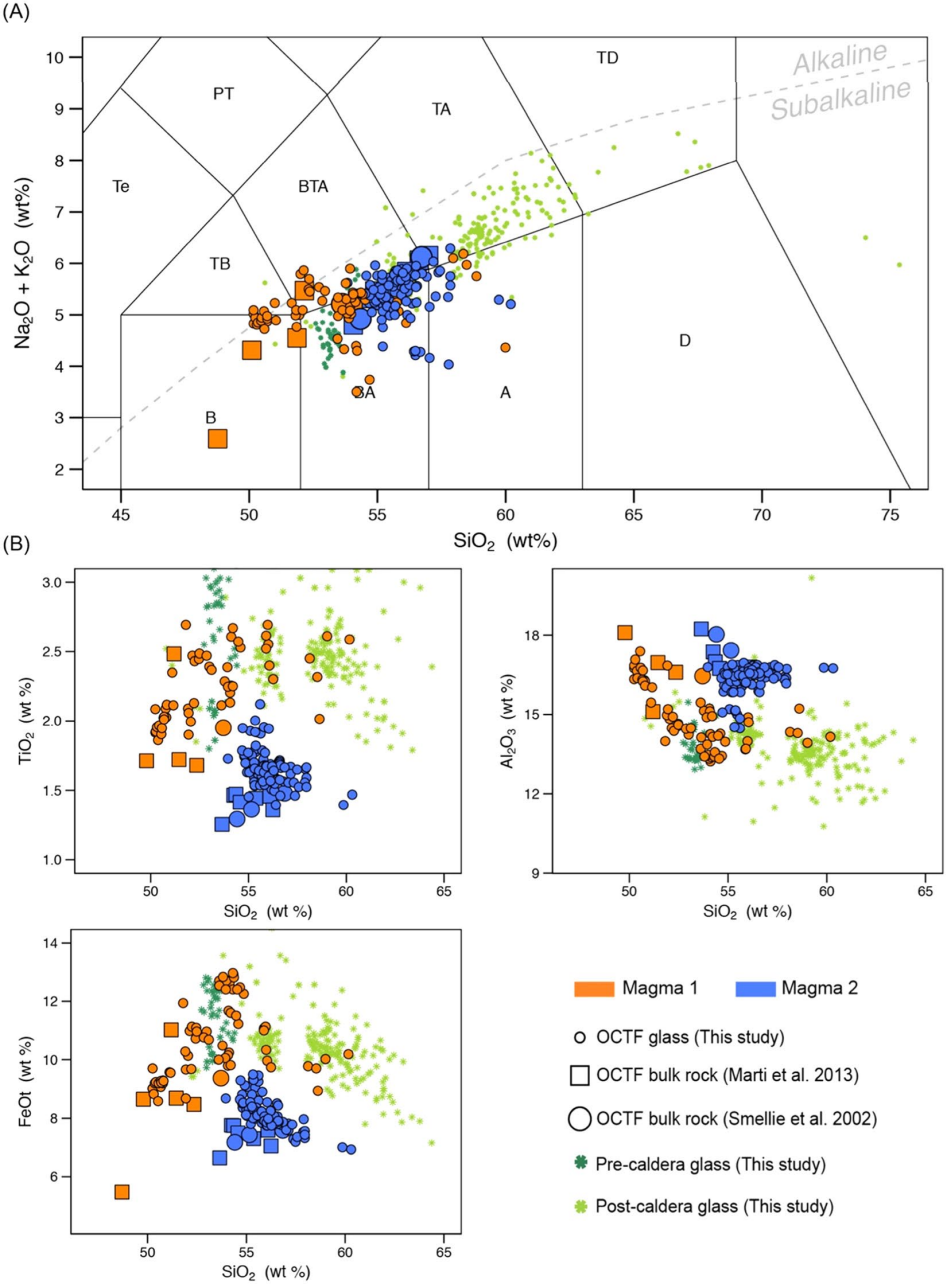
Glass and bulk rock magma composition of Deception Island’s pre-, post- and syn-caldera (OCTF) juvenile fragments. (**A**) Total Alkali vs. Silica diagram (TAS)^53^. Major elements are normalized to 100% (anhydrous) with Fe distributed between FeO and Fe_2_O_3_ following ref.^54^. The grey dashed line discriminates between the alkaline-subalkaline fields^55^. (**B**) Major element vs. SiO_2_ content Harker Diagrams. Major element compositions have been normalized to 100% in anhydrous base with Fe as FeOt. See Supplementary Information File 2 for details on composition and exact latitude-longitude coordinates of the rock samples. This figure was generated with RStudio Version 1.0.143 (https://www.rstudio.com/) using ggplot2 Version 2.1.9000. Final layout of this figure was achieved using Adobe Illustrator CC 2015.3.1 (Copyright © 1987–2016 Adobe Systems Incorporated and its licensors).

Several lines of evidence confirm that tephra T3 in Livingston Island lake sediments was produced by the Deception Island caldera-forming episode. On Deception Island, the Outer Coast Tuff Formation (OCTF; Fig. 2b) is accepted to be the stratigraphic unit that corresponds to the caldera-forming eruption. This formation, the most compositionally distinctive unit on Deception Island, is a sequence of pyroclastic density current deposits of mafic to intermediate composition^9,10,12^ (Fig. S2). The syn-caldera rocks contain evidence of mingling and co-eruption of two discrete magmas with geochemically distinct compositions, a feature found on Deception Island only in the OCTF. A first group of glass and bulk rock analyses of the OCTF fall within the main geochemical trend for Deception Island defined by the composition of pre- and post-caldera samples (Fig. 4 and Supplementary Fig. S2). By contrast, a second cluster groups at lower TiO_2_ and FeOt values, and for the same SiO_2_ have slightly higher concentrations of MgO, Al_2_O_3_ and CaO than the pre- and post-caldera rocks in major elements vs. SiO_2_ Harker diagrams (Fig. 4b and Supplementary Fig. S2). The presence of these two magmas suggests that the explosive eruption leading to caldera formation was triggered by the arrival of hotter and more primitive magmas into an existing reservoir^12^.

**Figure 5:**
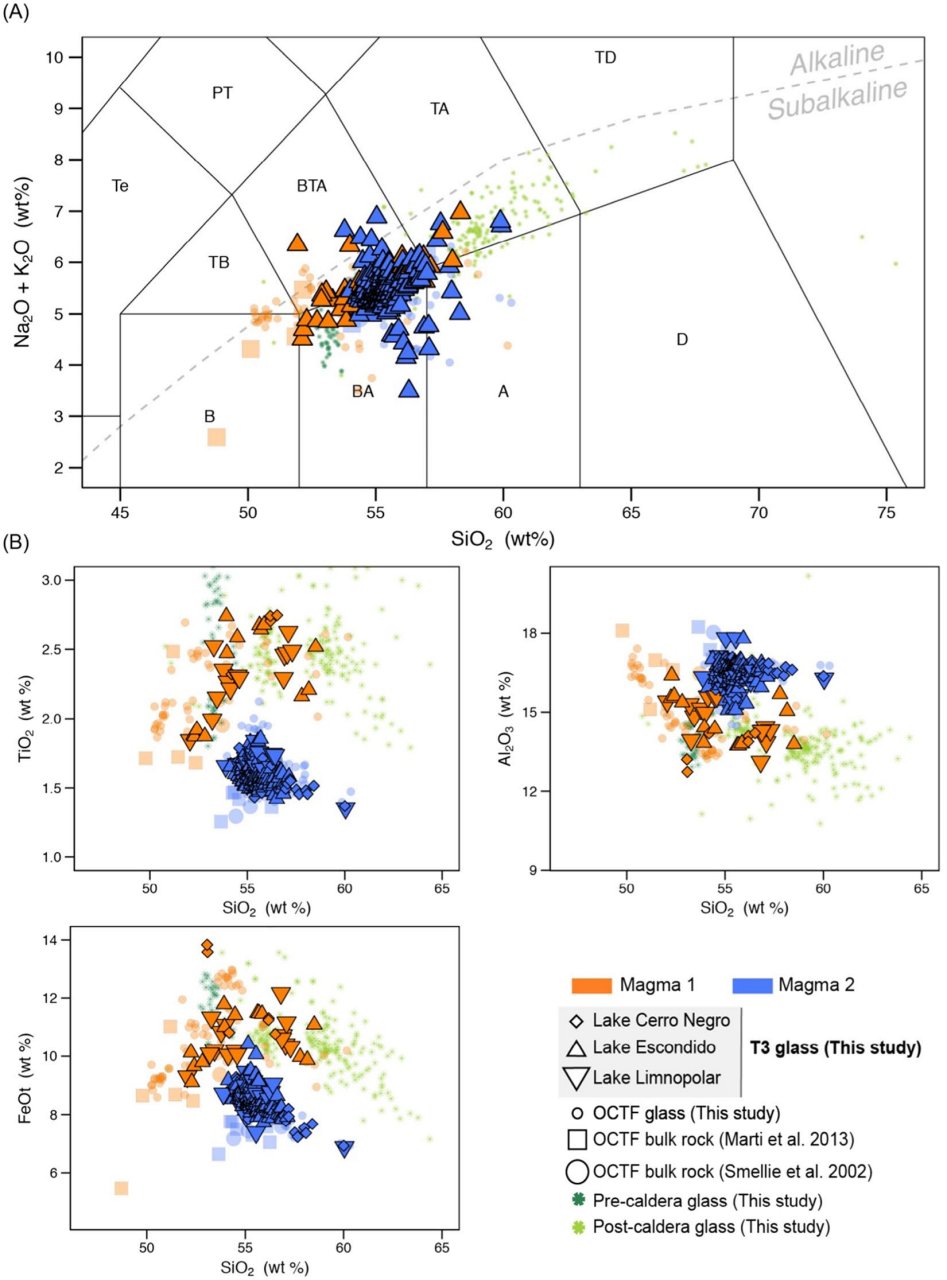
T3 glass compositions plotted over glass and bulk rock magma composition of Deception Island’s pre-, post- and syn-caldera (OCTF) juvenile fragments. (**A**) Total Alkali vs. Silica diagram (TAS)^53^. Major elements are normalized to 100% (anhydrous) with Fe distributed between FeO and Fe_2_O_3_ following ref.^54^. The grey dashed line discriminates between the alkaline-subalkaline fields^55^. (**B**) Major element vs. SiO_2_ content Harker Diagrams. Major element compositions have been normalized to 100% in anhydrous base with Fe as FeOt. See Supplementary Information File 2 for details on composition and exact latitude-longitude coordinates of the rock samples. This figure was generated with RStudio Version 1.0.143 (https://www.rstudio.com/) using ggplot2 Version 2.1.9000. Final layout of this figure was achieved using Adobe Illustrator CC 2015.3.1 (Copyright © 1987–2016 Adobe Systems Incorporated and its licensors).

Like the OCTF, the T3 tephra layer’s glass geochemistry also suggests the presence of two magma series (Fig. 5); when T3’s volcanic glasses are compared to those of the OCTF, there is a clear match of all major element relationships as well as locations on the Total Alkali Silica (TAS) diagram (Fig. 5 and Supplementary Fig. S3). Based on these correlations, we conclude that tephra T3 is analogous to the syn-caldera OCTF formation and therefore corresponds to distal fallout from the event shortly before or during the caldera collapse.

Magmatic temperatures and pressures calculated by applying silica-activity geothermobarometry to volcanic glasses (T1: 1094 ± 40 °C, 3.1 ± 0.2 kbar; T2: 1098 ± 40 °C, 3.2 ± 0.2 kbar; T3: 1110 ± 40 °C, 3.2 ± 0.2 kbar) closely reflect the basaltic-andesite source at *ca*. 10 km depth. Given that tephra T3 contains abundant microlite-poor juvenile sideromelane, while other tephra layers are characterised by more microlite-rich crystalline textures (juvenile tachylite^15^), we conclude that the magma ascent of T3 was rapid and without prolonged shallow storage and associated late-stage crystallisation prior to eruption. T3 is also the only tephra in Byers Peninsula lakes that contains juvenile clasts coarser than 2 mm in diameter, whose deposition here requires an eruption column tall enough to transport the coarse particles over 40 km against the prevailing wind direction. Their presence is thus indicative of a major eruptive event; the finer grain-size distributions (all particles <250 µm)^15^ characteristic of all other lacustrine tephra layers are consistent with much smaller eruptions.

Hydrogen (δD) and oxygen (δ^18^O) isotopes in ejecta record the modification of the original magmatic signal by interaction with meteoric fluids and seawater. The measured isotopic values from Byers Peninsula provide evidence for the pre-collapse characteristics of the T3 magma as well as the evolution of Deception Island magmas during subsequent eruptions (tephras T2 and T1; 2370 ± 100 and 1890 ± 140 cal y BP, respectively; Fig. 3). T3 δD (−51.2‰; Supplementary Fig. S4) reflects its equilibrium with primary magmatic fluids and indicates that the T3 eruption occurred prior to seawater infiltration into the magma chamber and therefore prior to caldera collapse. By contrast, the post-collapse tephra layers T2 and T1 are characterised by progressive δD shifts towards oceanic values (−26.3‰ and −4.4‰, respectively; Supplementary Fig. S4) that document the admixture of seawater into the evacuated magmatic system during subsequent volcanic episodes^24^. The minimal variation in δ^18^O between the three tephra layers (range: 5.6–6.1‰) is consistent with limited fractionation typical of such high-T magmas and the relatively small δ^18^O differences between magmatic and non-magmatic waters.

**Figure 6:**
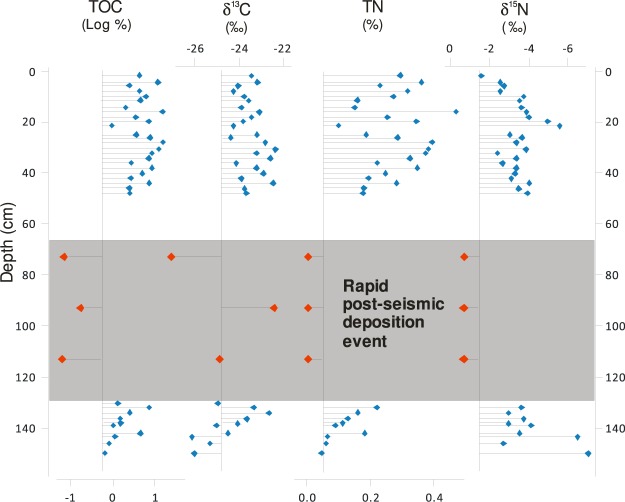
Sediment organic geochemical variables in Lake Escondido, Byers Peninsula. The shaded gray area represents the portion of the record with rapid terrestrial sedimentation. TOC, TN and δ^15^N all differ significantly (p < 0.05) between event sediments and those deposited during the pre- and post-event periods.

Whereas geochemical analyses record information about the eruptive process, the stratigraphic setting of lacustrine tephra provides insights into associated palaeoseismicity. Deposits up to 112 cm thick are immediately superposed upon T3 in each of the four studied Byers Peninsula lakes (Fig. 3). The genesis of these beds was determined by characterizing the temporal evolution of several depositional proxies, which included lithology, geochemistry, diatom assemblages and radiocarbon ages. These proxies showed that these were event beds that resulted from rapid mass-wasting of allochthonous sediments. Above and below the deposits, sediments consist of a centimetre-scale alternation of dark brown moss-rich layers and light brown massive lacustrine clays and silty clays. Strikingly different characteristics within the mass-wasting beds include massive lithology, an absence of mosses, and sediment geochemical properties. Radiocarbon ages immediately above and below the beds confirm their rapid deposition. Within the beds, however, anomalous radiocarbon dates that deviate several thousand years from the chronosequence reflect the ^14^C-depleted carbon associated with the redeposition of catchment material (Fig. 3). Marked differences in organic matter geochemistry provide further evidence for the terrestrial origin of the rapid post-seismic deposition sediments (Fig. 6). The differences in TOC, TN, and δ^15^N between Lake Escondido sediments within event beds and those of pre- and post-event sediments are significant (p < 0.05) and indicate shifts between long-term organic lacustrine deposition and values that more closely reflect the soils of nearby terrestrial environments^25^, providing parallel evidence of the catchment origin of the sediments in seismic event beds. Above the event deposits, geochemical characteristics returned to ‘typical’ lacustrine values.

Diatom analysis of the Lake Limnopolar sedimentary record clearly showed that the species present in the mass-wasted sediments were derived from terrestrial and aerophilic assemblages, while those before and after the event were lacustrine taxa. There were three distinct periods in Lake Limnopolar sediments: prior to (*Pre-Event*), during (*Event*) and following (*Post-Event*) the rapid sedimentation event. Diatom assemblages in *Pre-* and *Post-Event* sediments were dominated by taxa that are typical of lakes on the Byers Peninsula plateau, as expected with normal lacustrine deposition^26^. Interestingly, *Post-Event* assemblages (Supplementary Fig. S5) differed from *Event* diatoms but also from *Pre-Event* assemblages, which may indicate an ecological reorganisation due to the perturbation of algal communities by the eruption and its associated post-seismic event. In contrast to abundant deposition in *pre-* and *post-Event* sediments, diatoms were scarce in sediments laid down during the rapid deposition event; the minimum threshold for inclusion statistical analyses was not reached in all samples. In all cases, however, event samples were characterised by strikingly different assemblages than those in lacustrine sediments (Supplementary Fig. S5) and were dominated by species observed in soils, mosses and seepage samples in the Antarctic and sub-Antarctic regions (refs^26,27^, Table S2 and Supplementary Information).

Biological, geochemical and radiochronological data thus showed significant differences between the mass-wasted beds and adjacent sediments, indicating that event beds contained rapidly deposited terrestrial material. We propose that this deposition was triggered by an extreme seismic episode associated with the Deception Island caldera collapse. Distinctive mass-wasting deposits have been generated in lake sediments due to the extreme seismic activity that has accompanied other caldera collapse eruptions; analogous, meter-thick event beds are found in lakes hundreds of kilometres from the 1960 magnitude 9.5 Chilean earthquake, the largest ever recorded^23,28^. Numerous earthquakes up to Magnitude 7 were recorded in association with the Mt. Katmai caldera collapse in 1912 (caldera diameter 4 km)^23^ and, given its much greater size, it is reasonable to infer that seismic events of similar or superior magnitude were associated with the collapse of the Deception Island caldera. Seismic seiches, which have been observed in lakes located hundreds to thousands of kilometres from the epicentres of high magnitude earthquakes, are known to cause rapid post-seismic remobilisation of large volumes of terrestrial material from catchments to lake sediments, and are the likely mechanism for the deposition of the Byers Peninsula mass-wasting beds^28,29^.

To establish a rigorous chronology, we constrained the tephrostratigraphy with a total of 54 AMS ^14^C dates based on aquatic moss samples and three thermoluminescence ages (refs^30,31^, Fig. 3 and Supplementary Table 1). Bayesian age-depth models indicate that the caldera collapse eruption occurred 3980 cal y BP. We specifically avoided dating bulk sediment samples, given their well-demonstrated problems in providing reliable dates in Antarctic lake and ocean samples^14,32^, with the exception of samples taken within the rapid deposition event where no mosses were present. Twenty-nine radiocarbon dates encompassing five cores and three lakes (Escondido, Chester and Cerro Negro) were used to determine the age of the caldera collapse event based on calibrated ages immediately above the rapid sedimentation event and immediately below T3 (Fig. 3). Lake Escondido served as the basis for our chronology given the abundance of moss remains, the availability of suitable dating material immediately above and below collapse event sediments, and its robust age-depth model. In this lake, moss from 0.5 cm below T3 returned an age of 4060 ± 50 cal y BP, while moss 1.3 cm above the rapid deposition event was aged 3890 ± 50 cal y BP. Bayesian age-depth modelling further constrained this age, placing the caldera collapse at 3980 cal y BP, with a 95% confidence age range of 3860–4110 cal y BP^30^. Chester Lake sediments confirmed the timing of the caldera collapse, with moss radiocarbon ages in sediments above the termination of the rapid deposition event indicating that it occurred just prior to 3930 ± 25 cal y BP (Core CH12-0501G, 0.6 cm above event sediments), 3940 ± 30 cal y BP (Core CH12-0401; 9.4 cm above) and 3960 ± 50 cal y BP (Core CH12-0801; 0.5 cm above) (Fig. 3). Due to a lack of datable material, no older bounding age was available from Chester Lake; heavily folded sediments below the termination of the rapid deposition event and limited overlap between cores prevented the construction of a robust composite age model^30^.

In Lake Cerro Negro, the radiocarbon age nearest to event sediments was aged 3130 ± 50 cal y BP, however this age was 8.6 cm above the event’s termination. No material suitable for radiocarbon dating was found below this horizon, either above or below T3. Bayesian age-depth modelling indicated a minimum age for the caldera collapse event of 3635 ± 440 cal y BP, however the Lake Cerro Negro model was based on five moss ages (vs. 17 in the Escondido model) and before 3130 cal y BP was based entirely on a single thermoluminescence age^30^. Although the modelled collapse event age in Lake Cerro Negro is broadly consistent with those determined in the other cores, we gave primacy to the ages from lakes Escondido and Chester due to the stronger age model and the position of radiocarbon samples in stratigraphic proximity to event beds and tephra. Lake Limnopolar ages were not used to constrain the collapse event as our data suggest that they were subject to large and variable reservoir effect due to ancient carbon contamination most likely stored in catchment soils (see Supplementary Information for details).

Now firmly established as a Holocene event, the erupted volume of 30–60 km^3^ and volcanic explosivity index (VEI) of 6^9,10,12^ make the Deception Island caldera-forming eruption the largest eruption documented in Antarctica during the Holocene, exceeding the Hudson Mountains Subglacial Volcano (VEI 3-4; ref.^33^) by several orders of magnitude. Although an event of this magnitude would be expected to have widespread environmental consequences, until now, the lack of firm chronological constraint for the Deception Island caldera collapse had precluded an evaluation of the scope of its impacts. We therefore examined Antarctic ice cores, and lacustrine and marine sediments throughout the SSI and the Antarctic Peninsula, in search of contemporaneous volcanogenic layers and comparable event beds, revealing at least 18 such sites around Antarctica that preserve a record of the caldera collapse eruption (Fig. 1 and Supplementary Table 3). The distribution suggests that volcanic ejecta were transported by a predominantly westerly airstream and deposited across a wide region of the sub-Antarctic, with deflection onto the Antarctic continent mediated by the polar vortex, as indicated by modelling of recent Deception Island eruptions^34^. Glaciochemical signatures record a major volcanic event in Antarctic ice cores reaching >4600 km from Deception Island, with modelled ice ages that closely matched the Deception Island caldera-forming event. These included Talos Dome (3998 ± 130 y BP); Dome C (4004 ± 200 y BP); Dronning Maud Land (3995 ± 200 y BP); and Vostok (3942 ± 600 y BP)^35–40^, as well as Plateau Remote (3969 ± 100 y BP)^41^, whilst a tephra tentatively aged 3910 ± 200 y BP was deposited in a James Ross Island ice core^18^, although the correlations remain tenuous until they can be confirmed by geochemical composition data for these tephra. Tephra layers that we correlated to the Deception Island caldera collapse by a combination of chronology and/or geochemistry were also found in sediment cores and terrestrial sites situated across the SSI, James Ross Island and the Antarctic Peninsula, as well as in palaeoceanographic records from Bransfield Strait and the Scotia Sea (Fig. 1 and Supplementary Information). Several lakes from the Barton, Fildes and Potter peninsulas of King George Island, ~120–130 km downwind of Deception Island, contain eruptive-seismic depositional sequences with tephra, gravity flows and rapid-deposition events that in some instances reach 1.5 m thickness (Fig. 1 and Supplementary Table S3; refs^42,43^). The sedimentary record of another two King George Island lakes record tephra and periods of accelerated sedimentation, combined with the deposition of terrestrial diatoms and reductions in organic matter content due to inputs of allochthonous material, that are consistent with our event chronology^44^. These singular, largely unexplained stratigraphic features mirror those on Livingston Island and underline the magnitude of both the Deception Island eruption and the seismic episode.

Taken together, our study reveals conclusive, multidisciplinary evidence for a major volcanic episode with far-reaching effects at 3980 cal y BP. Ejecta from the caldera-forming eruption were deposited widely across the Antarctic, whilst a major coeval earthquake affected the Antarctic Peninsula region. In addition to resolving the long-lasting controversy regarding the timing of this major volcanic episode, these findings provide a valuable tie point for the calibration of ice and sediment core chronologies and will therefore contribute to an improved understanding of past Antarctic environmental change. Much remains to be elucidated about the effects of large eruptions on climate, and the data available at present are insufficient to provide a detailed characterisation of the climatic and ecological response to this and other profound but short-lived Antarctic events^1,7^. However, our study establishes a precise chronology for the Deception Island caldera collapse, a volcano-climatic event with a likely hemispheric impact. Future, high-resolution re-examination of ice and sediment records should help to clarify the influence of such volcanic forcing on Antarctic climate.

## Methods

### Sites, coring and subsampling

During Austral summer 2008 (Limnopolar Lake, 62° 37′ 23.65″S, 61° 06′ 23.67″W) and 2012 (Escondido Lake, 62° 37′ 06.57″S, 61° 03′ 36.51″W; Chester Lake, 62° 36′ 41.10″S, 61° 06′ 02.28″W; and Cerro Negro Lake, 62° 37′ 47.30″S, 61° 00′ 19.99″W) lakes were cored until the underlying glacial till was reached using a 90 mm diameter UWITEC piston corer mounted on a tripod as well as a 60 mm diameter UWITEC gravity corer to recover the undisturbed sediment-water interface, using the lake ice covers as platforms. Limnopolar Lake sediments were extruded, cut in 60 cm sections, and sealed for shipping, whilst the sediments of the other four lakes were sealed for shipping directly in the plastic core liners. All cores were stored in a cold room at 4 °C until they were opened in the laboratory, when they were split longitudinally and subsamples were taken from the centres of cores.

### Geochronology

Stratigraphic ages were determined throughout cores in order to understand overall trends in sedimentation. In order to constrain event chronologies, particular attention was paid to samples immediately above and below tephra and the rapid, deposition event that was clearly distinguishable from the otherwise regular alternation of organic and mineral sedimentation. Fifty-four AMS radiocarbon measurements were performed, almost exclusively on large moss fragments, with samples from Limnopolar dated at the Poznań Radiocarbon Lab (Poznań, Poland) and those from the other four lakes prepared at the Université Laval Laboratoire de Radiochronologie (Quebec, Canada) and measured at the Keck Carbon Cycle Accelerator Mass Spectrometry Laboratory (Irvine, USA), respectively, along with three thermoluminescence ages measured at the base of cores in order to corroborate ^14^C data^30,31^. All radiocarbon ages were calibrated using the SHCal13 curve for the Southern Hemisphere^45^ and are listed in calibrated years before present (cal y BP), with the present indicating the year 1950 AD. A full list of ^14^C samples, accession numbers and data as well as other details about the chronology can be found in the Supplementary Information.

### Bulk organic geochemistry and sediment stable isotope analysis

Samples were taken throughout two cores for sediment organic matter (total organic carbon: TOC; and total nitrogen: TN) and stable isotope analysis of N (δ^15^N) and C (δ^13^C). Samples were dried at 60 °C for 48 h and ground by hand using an agate mill. TOC, TN, δ^13^C and δ^15^N were determined using a Finnigan DELTAplus elemental analyser-continuous flow-isotope ratio mass spectrometer (EA-CF-IRMS) at the Centres Científics i Tecnològics of the Universitat de Barcelona (CCiTUB). The relative standard deviation (RSD) for TOC and TN measurements was 5% whilst the analytical precision for δ^13^C and δ^15^N was 0.2‰. Stable isotope ratios are reported in δ notation using parts per thousand units (‰) and are defined as δ = [(R_sample_/R_standard_) − 1] × 1000.

### Tephra geothermobarometry, geochemistry and stable isotope analysis

Magmatic temperatures and pressures were calculated by applying silica-activity geothermobarometry to volcanic glasses. Because the water content was not constrained, we assigned an H_2_O content range from 1 to 4%, just sufficient to achieve saturation, and utilised the silica-activity (in glass) geothermobarometer of Albarède^46^, which precisely matches with the formation conditions of basalt-andesite shield-volcanoes. Water variations gave negligible P-T fluctuations.

The composition of tephra samples from T1, T2, and T3 was determined by electron microprobe analysis at the Centres Científics i Tecnològics Universitat de Barcelona and the University of Bristol. Byers Peninsula tephra samples (0.5–2 g) were cleaned in an ultrasonic bath for five minutes and dried overnight at 80 °C. Dried samples were then sieved into individual size fractions at phi (φ) intervals from −1φ to <3φ (corresponding to >2 mm, 1–2 mm, 500–1000 µm, 250–500 µm, 125–250 µm, and <125 µm; φ = −log_2_d, where d is the particle diameter in mm). Matrix glass major element compositions (as oxides) were measured on carbon-coated polished grain mounts of the 0–1φ, 1–2φ, and 2–3φ size fractions using the Cameca SX100 electron microprobe at the University of Bristol. Analyses were performed under operating conditions of 15 kV accelerating voltage and 4 nA beam current using a defocused beam (10 μm) to minimise sodium mobility. A combination of mineral and glass standards were used for calibration. Typical 1σ standard deviation of counts was between 0.5 and 1%. Analyses of secondary standards (KK1, BCR2 and diopside) were repeated each day to identify any instrumental drift, for which no corrections were necessary. Counting times were 10 s for Na, Si, Al, K and Ca, and 30 s for Mg, S, P, Ti, Fe and Mn, over a total analysis time of 60 s. A total of 40–50 glassy crystal-poor grains within the 125–250 µm (2–3φ) size fraction was analysed for each tephra layer, with each grain analysed once. Analyses that were contaminated by crystal phases were excluded. All compositions are shown normalised to anhydrous composition.

Oxygen isotope analyses were performed on whole-rock powders by laser fluorination^47^, using a Synrad 25 W CO_2_ laser^48^ and ClF_3_ as reagent (*cf*. ref.^49^). Isotope ratios were measured on a VG-Isotech SIRA-II dual-inlet mass spectrometer at the Servicio de Isótopos Estables (Nucleus, University of Salamanca). The preparation systems included both a conventional and a laser fluorination line (δ^18^O determination in silicates), a D/H high-vacuum line for hydrogen extraction from hydrated minerals with a uranium-depleted reduction system, and a step-heating device for the fractional extraction and purification of fluids (liquid, gas) from potential presence of glass inclusions. Both internal and international reference standards (NBS-28, NBS-30) were run to check accuracy and precision. Results are reported in δ^18^O notation relative to the Vienna Standard Mean Ocean Water (V-SMOW) standard, using a δ^18^O value of 9.6‰ for NBS-28 (quartz) for the mass spectrometer calibration. Long-term reproducibility for repeated determination of reference samples was better than ± 0.2‰ (1σ).

D/H ratios were determined on a second SIRA-II mass spectrometer on H_2_ gas obtained by reduction over hot depleted-U of the water released by induction heating of samples, using a vacuum line, following the procedures described in Godfrey^50^ with modifications^51^. Samples were loaded into degassed platinum crucibles that were placed in quartz reaction tubes and heated under vacuum to 125 °C overnight to remove any adsorbed H_2_O. The yield of evolved gas was used to determine the amount of structural water contained in the sample. Results are reported in δD notation relative to the V-SMOW standard, using a δD = −66.7‰ for NBS-30 (biotite) for the mass spectrometer calibration. Long-term reproducibility for repeated determination of reference samples was better than ± 2‰ (1σ).

### Diatoms

Samples for diatom analysis were taken at regular intervals from Lake Limnopolar (core LIM08). Organic matter was oxidised by treatment with hydrogen peroxide (33% H_2_O_2_) and samples were mounted in Naphrax following methods described in ref.^27^. Three hundred diatom valves from each sample were identified and enumerated on random transects at 1000x magnification under oil immersion using a Zeiss Axio Imager A1 microscope equipped with differential interference contrast optics. See Supplementary information for further details about diatom identification and statistical analyses.

## Electronic supplementary material

Supplementary Information

Supplementary Dataset 1

## Data Availability

The tephra datasets generated during and/or analysed during the current study are available in Supplementary Information File 2.

## References

[1] Robock A (2000). Volcanic eruptions and climate. Rev. Geophys..

[2] Oppenheimer C (2015). Eruption politics. Nat. Geosci..

[3] Büntgen U (2016). Cooling and societal change during the Late Antique Little Ice Age from 536 to around 660 AD. Nat. Geosci..

[4] Sigl M (2015). Timing and climate forcing of volcanic eruptions for the past 2,500 years. Nature.

[5] Pausata FSR, Chafik L, Caballero R, Battisti DS (2015). Impacts of high-latitude volcanic eruptions on ENSO and AMOC. Proc. Natl. Acad. Sci. USA.

[6] Oman, L., Robock, A., Stenchikov, G., Schmidt, G. A. & Ruedy, R. Climatic response to high‐latitude volcanic eruptions. *J. Geophys. Res.-Atmos*. **110**(**D13**) (2005).

[7] Sigl M (2016). Insights from Antarctica on volcanic forcing during the Common Era. Nat. Clim. Change.

[8] Smellie JL (1999). The upper Cenozoic tephra record in the south polar region: a review. Global Planet. Change.

[9] Smellie JL (2001). Lithostratigraphy and volcanic evolution of Deception Island, South Shetland Islands. Antarct. Sci..

[10] Martí J, Geyer A, Aguirre-Diaz G (2013). Origin and evolution of the Deception Island caldera (South Shetland Islands, Antarctica). Bull. Volcanol..

[11] Kandlbauer J, Sparks RSJ (2014). New estimates of the 1815 Tambora eruption volume. J. Volcanol. Geoth. Res..

[12] Smellie, J. L. *et al*. *Geology and geomorphology of Deception Island* (British Antarctic Survey, 2002).

[13] Oliva-Urcia B (2015). Paleomagnetism from Deception Island (South Shetlands archipelago, Antarctica), new insights into the interpretation of the volcanic evolution using a geomagnetic model. Int. J. Earth Sci..

[14] Björck S, Sandgren P, Zale R (1991). Late Holocene tephrochronology of the northern Antarctic Peninsula. Quaternary Res..

[15] Liu EJ (2016). Expanding the tephrochronological framework for Byers Peninsula, Antarctica, by combined compositional and textural fingerprinting. Sediment. Geol..

[16] Moreton SG, Smellie JL (1998). Identification and correlation of distal tephra layers in deep-sea sediment cores, Scotia Sea, Antarctica. Ann. Glaciol..

[17] Narcisi B, Petit JR, Delmonte B, Basile-Doelsch I, Maggi V (2005). Characteristics and sources of tephra layers in the EPICA-Dome C ice record (East Antarctica): implications for past atmospheric circulation and ice core stratigraphic correlations. Earth Planet. Sc. Lett..

[18] Mulvaney R (2012). Recent Antarctic Peninsula warming relative to Holocene climate and ice-shelf history. Nature.

[19] Bartolini S, Geyer A, Martí J, Pedrazzi D, Aguirre-Díaz G (2014). Volcanic hazard on Deception Island (South Shetland Islands, Antarctica). J. Volcanol. Geoth. Res..

[20] Almendros J, Carmona E, Jiménez V, Díaz-Moreno A, Lorenzo F (2018). Volcano-tectonic activity at Deception Island Volcano following a seismic swarm in the Bransfield Rift (2014–2015). Geophys. Res. Lett..

[21] Moreton, S. G. Quaternary tephrochronology of the Scotia Sea and Bellingshausen Sea, Antarctica. Ph.D. thesis, Cheltenham and Gloucester College of Higher Education, 164 pp. (1999).

[22] Roberts Stephen J., Monien Patrick, Foster Louise C., Loftfield Julia, Hocking Emma P., Schnetger Bernhard, Pearson Emma J., Juggins Steve, Fretwell Peter, Ireland Louise, Ochyra Ryszard, Haworth Anna R., Allen Claire S., Moreton Steven G., Davies Sarah J., Brumsack Hans-Jürgen, Bentley Michael J., Hodgson Dominic A. (2017). Past penguin colony responses to explosive volcanism on the Antarctic Peninsula. Nature Communications.

[23] Hildreth, W. & Fierstein, J. The Novarupta-Katmai eruption of 1912—largest eruption of the twentieth century: centennial perspectives. *U.S. Geol. Surv. Prof. Paper* **1791** (2012).

[24] Valley, J. W. & Cole, D. (Eds). Stable isotope geochemistry. *Rev. Mineral. Geochem*. **43** (2001).

[25] Otero XL, Fernández S, de Pablo Hernandez MA, Nizoli EC, Quesada A (2013). Plant communities as a key factor in biogeochemical processes involving micronutrients (Fe, Mn, Co, and Cu) in Antarctic soils (Byers Peninsula, maritime Antarctica). Geoderma.

[26] Kopalová K, Van de Vijver B (2013). Structure and ecology of freshwater benthic diatom communities from Byers Peninsula, Livingston Island, South Shetland Islands. Antarct. Sci..

[27] Pla-Rabes S (2013). Stability and endemicity of benthic diatom assemblages from different substrates in a maritime stream on Byers Peninsula, Livingston Island, Antarctica: the role of climate variability. Antarct. Sci..

[28] Van Daele M (2015). A comparison of the sedimentary records of the 1960 and 2010 great Chilean earthquakes in 17 lakes: Implications for quantitative lacustrine palaeoseismology. Sedimentology.

[29] McGarr, A. & Vorhis, R. C. Seismic seiches from the March 1964 Alaska earthquake. *U.S. Geol. Surv. Prof. Paper* **544-E** (1968).

[30] Oliva M (2016). The Holocene deglaciation of the Byers Peninsula (Livingston Island, Antarctica) based on the dating of lake sedimentary records. Geomorphology.

[31] Toro M (2013). Chronostratigraphy of the sedimentary record of Limnopolar Lake, Byers Peninsula, Livingston Island, Antarctica. Antarct. Sci..

[32] Moreton SG, Rosqvist GC, Davies SJ, Bentley MJ (2004). Radiocarbon reservoir ages from freshwater lakes, South Georgia, sub-Antarctic: modern analogues from particulate organic matter and surface sediments. Radiocarbon.

[33] Corr HFJ, Vaughan DG (2008). A recent volcanic eruption beneath the West Antarctic ice sheet. Nat. Geosci..

[34] Geyer A, Martí A, Giralt S, Folch A (2017). Potential ash impact from Antarctic volcanoes: insights from Deception Island’s most recent eruption. Sci. Rep. UK.

[35] Severi M, Udisti R, Becagli S, Stenni B, Traversi R (2012). Volcanic synchronisation of the EPICA-DC and TALDICE ice cores for the last 42 kyr BP. Clim. Past.

[36] Veres D (2013). The Antarctic ice core chronology (AICC2012): an optimized multi-parameter and multi-site dating approach for the last 120 thousand years. Clim. Past.

[37] Castellano E (2005). Holocene volcanic history as recorded in the sulfate stratigraphy of the European Project for Ice Coring in Antarctica Dome C (EDC96) ice core. J. Geophys. Res.-Atmos..

[38] Ruth U (2007). EDML1: a chronology for the EPICA deep ice core from Dronning Maud Land, Antarctica, over the last 150 000 years. Clim. Past.

[39] Severi M (2007). Synchronisation of the EDML and EDC ice cores for the last 52 kyr by volcanic signature matching. Clim. Past.

[40] Udisti R (2004). Stratigraphic correlations between the European Project for Ice Coring in Antarctica (EPICA) Dome C and Vostok ice cores showing the relative variations of snow accumulation over the past 45 kyr. J. Geophys. Res.-Atmos..

[41] Cole-Dai J, Mosley-Thompson E, Wight SP, Thompson LG (2000). A 4100-year record of explosive volcanism from an East Antarctica ice core. J. Geophys. Res.-Atmos..

[42] Tatur A, del Valle AR, Pazdur M (1991). Lake sediments in maritime Antarctic zone: a record of landscape and biota evolution: preliminary report. Int. Ver. The..

[43] Lee YI, Lim H-S, Yoon HI, Tatur A (2007). Characteristics of tephra in Holocene lake sediments on King George Island, West Antarctica: implications for deglaciation and paleoenvironment. Quaternary Sci. Rev..

[44] Schmidt R, Mäusbacher R, Müller J (1990). Holocene diatom flora and stratigraphy from sediment cores of two Antarctic lakes (King George Island). J. Paleolimnol..

[45] Hogg AG (2013). SHCal13 Southern Hemisphere calibration, 0–50,000 years cal BP. Radiocarbon.

[46] Albarède F (1992). How deep do common basaltic magmas form and differentiate?. J. Geophys. Res. - Sol. Ea..

[47] Clayton RN, Mayeda TK (1963). The use of bromine pentafluoride in the extraction of oxygen from oxides and silicates for isotopic analysis. Geochim. Cosmochim. Ac..

[48] Sharp ZD (1990). A laser-based microanalytical method for *in situ* determination of oxygen isotope ratios of silicates and oxides. Geochim. Cosmochim. Ac..

[49] Borthwick J, Harmon RS (1982). A note regarding ClF_3_ as an alternative to BrF_5_ for oxygen isotope analysis. Geochim. Cosmochim. Ac..

[50] Godfrey JD (1962). The deuterium content of hydrous minerals from the east-central Sierra Nevada and Yosemite National Park. Geochim. Cosmochim. Ac..

[51] Jenkin, G. R. T. Stable isotope studies in Caledonides of SW Connemara, Ireland. PhD thesis, Univ. Glasgow, UK (1988).

[52] Torrecillas, C., Berrocoso, M. & García-García, A. The multidisciplinary scientific information support system (SIMAC) for Deception Island [Fütterer, D. K., Damaske, D., Kleinschmidt, G., Miller, H. & Tessensohn, F. eds)] Antarctica: contributions to global earth sciences, 397–402 (Springer, 2006).

[53] Le Bas MJ, Le Maitre RW, Streckeisen A, Zanettin B (1986). A Chemical Classification of Volcanic Rocks Based on the Total Alkali-Silica Diagram. J. Petrol..

[54] Middlemost EAK (1989). Iron oxidation ratios, norms and the classification of volcanic rocks. Chem. Geol..

[55] Irvine TN, Baragar WRA (1971). A guide to the chemical classification of the common volcanic rocks. Can. J. Earth Sci..

