# Supplementary material for: The timing and widespread effects of the largest Holocene volcanic eruption in Antarctica
Source: Sci Rep. 2018 Nov 22;8:17279. doi: 10.1038/s41598-018-35460-x (PMC6250685; doi:10.1038/s41598-018-35460-x)
Supplement: Supplementary file 1 — Supplementary Information [file 41598_2018_35460_MOESM1_ESM.pdf]

Supplementary Information 1

The timing and widespread effects of the largest Holocene volcanic eruption in Antarctica

Dermot Antoniades[1*], Santiago Giralt[2], Adelina Geyer[2], Antonio M. Álvarez-Valero[3], Sergi Pla-Rabes[4,5], Ignacio Granados[6], Emma J. Liu[7], Manuel Toro[8], John L. Smellie[9] and Marc Oliva[10]

[1]*Department of Geography, Centre for Northern Studies & Takuvik Unité Mixte Internationale, Université Laval, G1V 0A6, Quebec, Canada.*

[2]*Institute of Earth Sciences Jaume Almera, ICTJA-CSIC, 08028 Barcelona, Spain*

[3]*Departamento de Geología, Universidad de Salamanca, 37008 Salamanca, Spain*

[4]*CREAF, 08193 Cerdanyola del Vallès, Spain*

[5] *Departament de Biociències, UST- Facultat de Ciències i Tecnologia Universitat de Vic-Universitat Central de Catalunya, 0850, Spain*

[6]*Centro de Investigación, Seguimiento y Evaluación, Sierra de Guadarrama National Park, 28740 Rascafría, Spain*

[7]*Department of Earth Sciences, University of Cambridge, Downing Street, Cambridge, CB2 3EQ, U.K.*

[8]*Centre for Hydrographic Studies (CEDEX), 28005 Madrid, Spain*

[9]*Department of Geology, University of Leicester, Leicester, LE1 7RH, U.K.*

[10]*Department of Geography, Universitat de Barcelona, 08001 Barcelona, Spain*

[*]Corresponding author: Department of Geography, Centre for Northern Studies & Takuvik Unité Mixte Internationale, Université Laval, G1V 0A6 Quebec, Canada. +1 418-656-2131 ext. 8996; dermot.antoniades@cen.ulaval.ca.

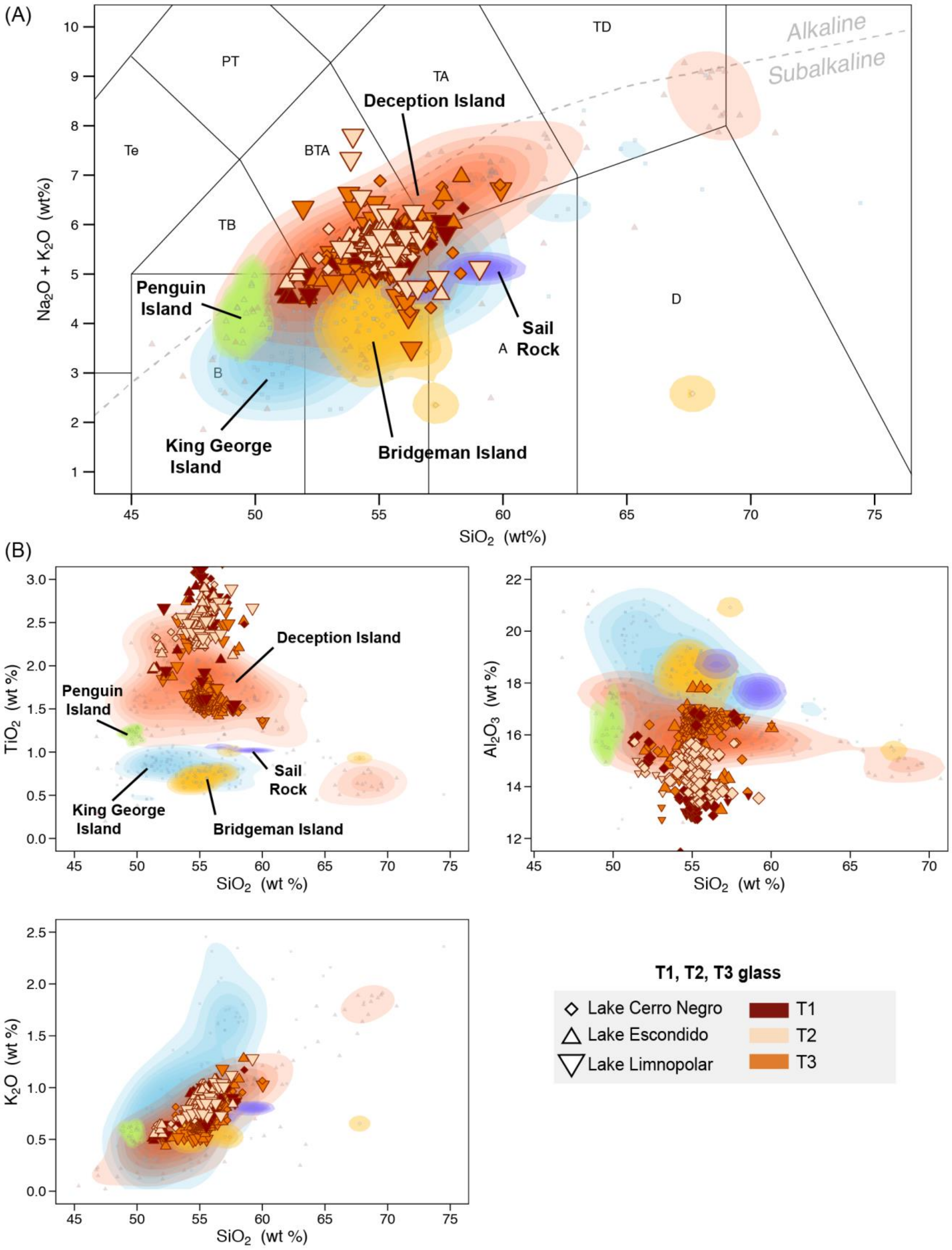
(A)
Alkaline
Subalkaline
TD
PT
TA
Deception Island
Te
BTA
TB
Penguin Island
Sail Rock
D
A
B
King George Island
Bridgeman Island
$Na_2O + K_2O$ (wt%)
SiO_2 (wt%)
(B)
Deception Island
Penguin Island
Sail Rock
King George Island
Bridgeman Island
TiO_2 (wt %)
Al_2O_3 (wt %)
K_2O (wt %)
SiO_2 (wt %)
T1, T2, T3 glass
Lake Cerro Negro
Lake Escondido
Lake Limnopolar
T1
T2
T3

Fig. S1. Correlating the glass compositions source of the three tephra samples T1,T2 and T3 of Byers Peninsula within the compositional fields (bulk rock) of nearby volcanic centres, including Penguin Island (green), King George Island (blue), Deception Island (red), Bridgeman Island (yellow) and Sail Rock (lilac). (A) Total Alkali vs. Silica diagram (TAS) (ref. 53). Major elements normalized to 100% (anhydrous) with Fe distributed between FeO and Fe_2O_3 following ref. 54. Grey dashed line discriminates between the alkaline-subalkaline fields[55]. (B) Major elements vs. SiO_2 content Harker Diagrams. Major element compositions have been normalized to 100% in anhydrous base with Fe as FeOt. See Supplementary Information 2 for details on composition and latitude-longitude coordinates of the rock samples. This figure was generated with RStudio Version 1.0.143 (https://www.rstudio.com/) using ggplot2 Version 2.1.9000[77]. Final layout of this figure was achieved using Adobe Illustrator CC 2015.3.1 (Copyright © 1987–2016 Adobe Systems Incorporated and its licensors).

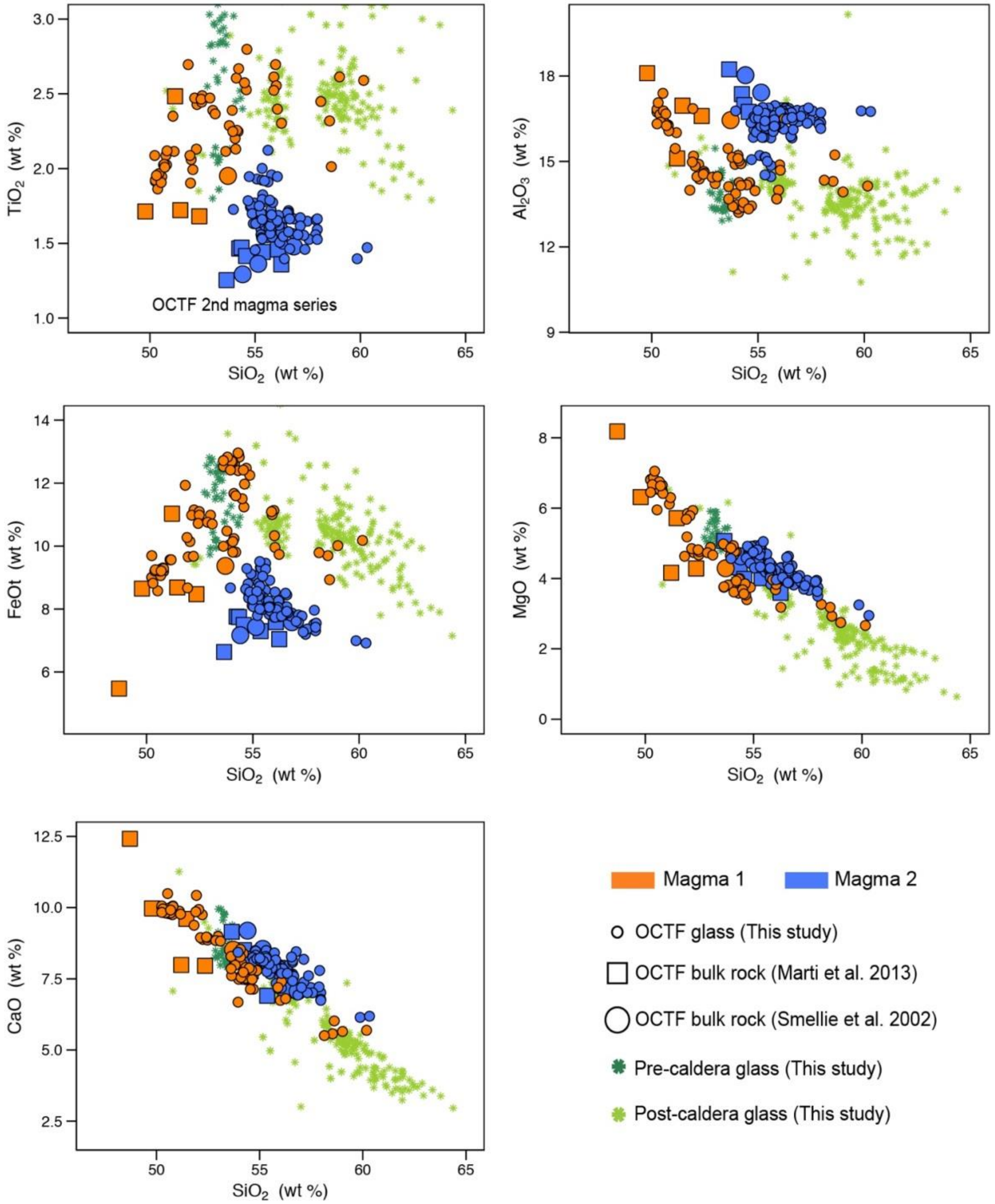
OCTF 2nd magma series
TiO_2 (wt %)
SiO_2 (wt %)
Al_2O_3 (wt %)
FeOt (wt %)
MgO (wt %)
CaO (wt %)
Magma 1
Magma 2
OCTF glass (This study)
OCTF bulk rock (Marti et al. 2013)
OCTF bulk rock (Smellie et al. 2002)
Pre-caldera glass (This study)
Post-caldera glass (This study)

Fig. S2. Harker diagrams showing magma composition (glass and bulk rock, normalized to 100 %) of Deception Island pre-, post- and syn-caldera (OCTF) juvenile fragments. Compositions belonging to the 1st and 2nd OCTF magma series are in orange and blue, respectively. The location of the collected samples is indicated in Supplementary file 2. This figure was generated with RStudio Version 1.0.143 (https://www.rstudio.com/) using ggplot2 package Version 2.1.9000[77]. Final layout of this figure was achieved using Adobe Illustrator CC 2015.3.1 (Copyright © 1987–2016 Adobe Systems Incorporated and its licensors).

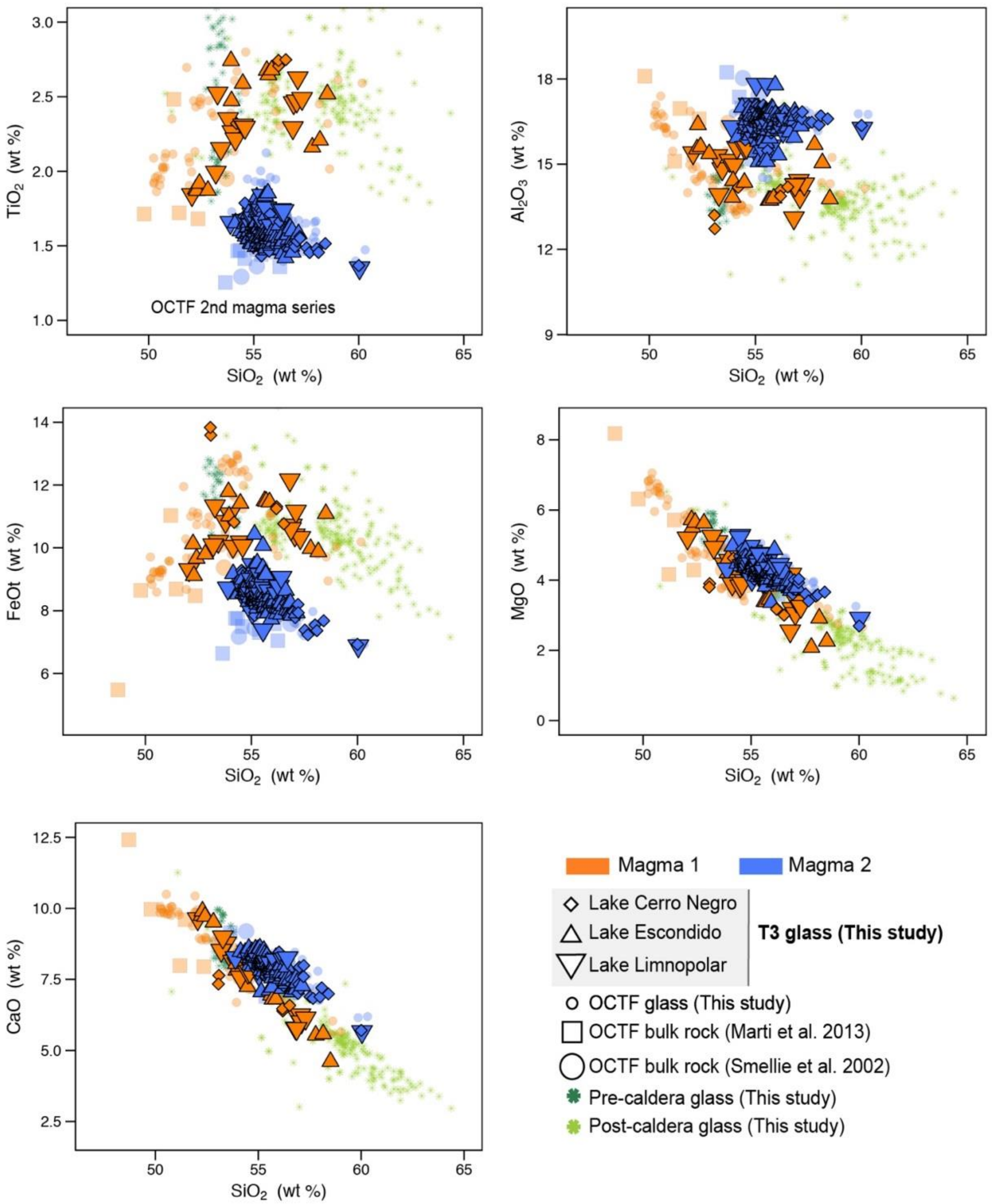
OCTF 2nd magma series
TiO2 (wt %)
Al2O3 (wt %)
FeOt (wt %)
MgO (wt %)
CaO (wt %)
SiO2 (wt %)
Magma 1
Magma 2
Lake Cerro Negro
Lake Escondido
Lake Limnopolar
T3 glass (This study)
OCTF glass (This study)
OCTF bulk rock (Marti et al. 2013)
OCTF bulk rock (Smellie et al. 2002)
Pre-caldera glass (This study)
Post-caldera glass (This study)

Fig. S3. Harker diagrams showing magma composition (glass and bulk rock, normalized to 100 %) of Deception Island pre-, post- and syn-caldera (OCTF) juvenile fragments collected during the austral summer field work of 2010-2011 and 2012-2013. T3 juvenile glass fragments analysed from lake tephra layers in this study are also included. Compositions belonging to the 1st and 2nd OCTF magma series are in orange and blue, respectively. The location of the collected samples is indicated in Supplementary file 2. This figure was generated with RStudio Version 1.0.143 (https://www.rstudio.com/) using ggplot2 package Version 2.1.9000[77]. Final layout of this figure was achieved using Adobe Illustrator CC 2015.3.1 (Copyright © 1987–2016 Adobe Systems Incorporated and its licensors).

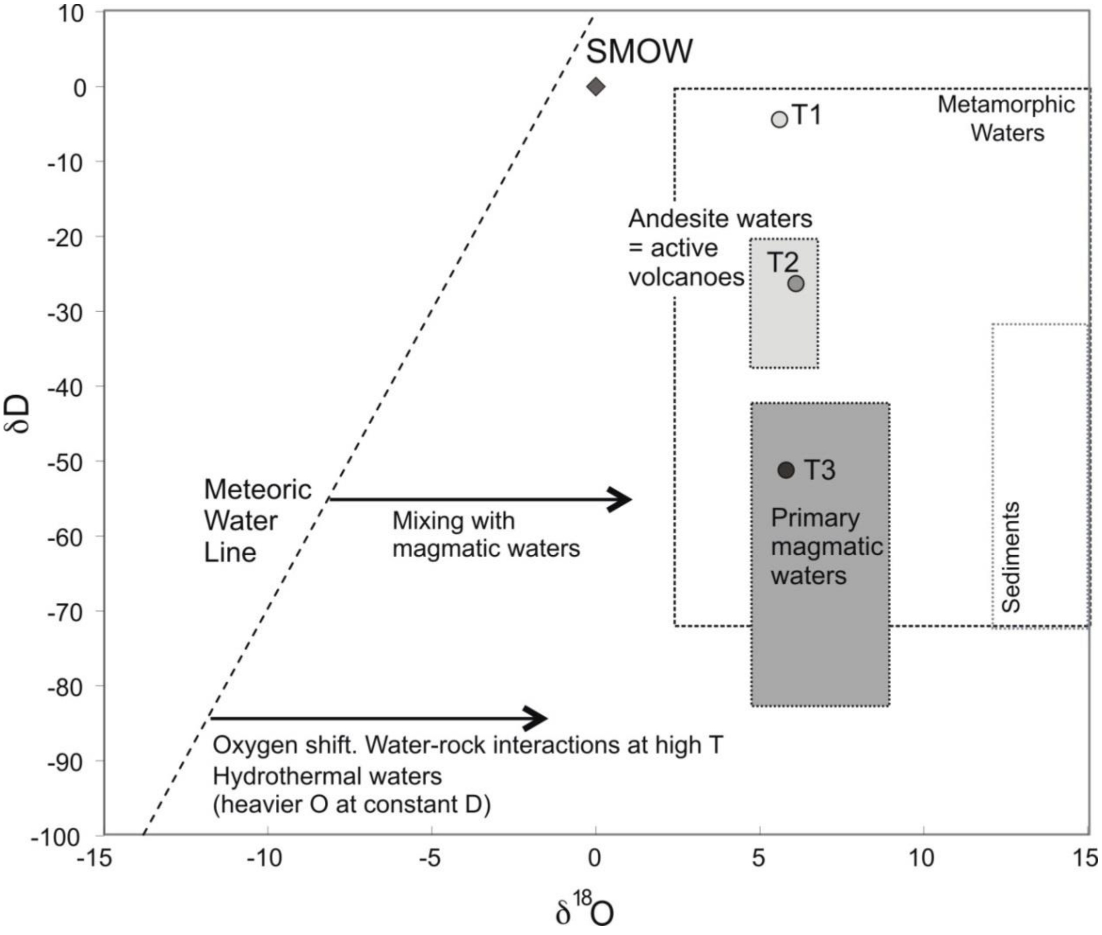

Fig. S4. Representation of δD vs. $\delta^{18}O$ values (‰) measured in Deception Island tephra from Byers Peninsula in Taylor's diagram (ref. 56) of water isotopic composition. The temperature of the magmas (ca. 1100 ºC) did not allow for oxygen equilibration or fractionation (typical at such high T) during rapid eruption, and water δD values are therefore comparable with solid samples.

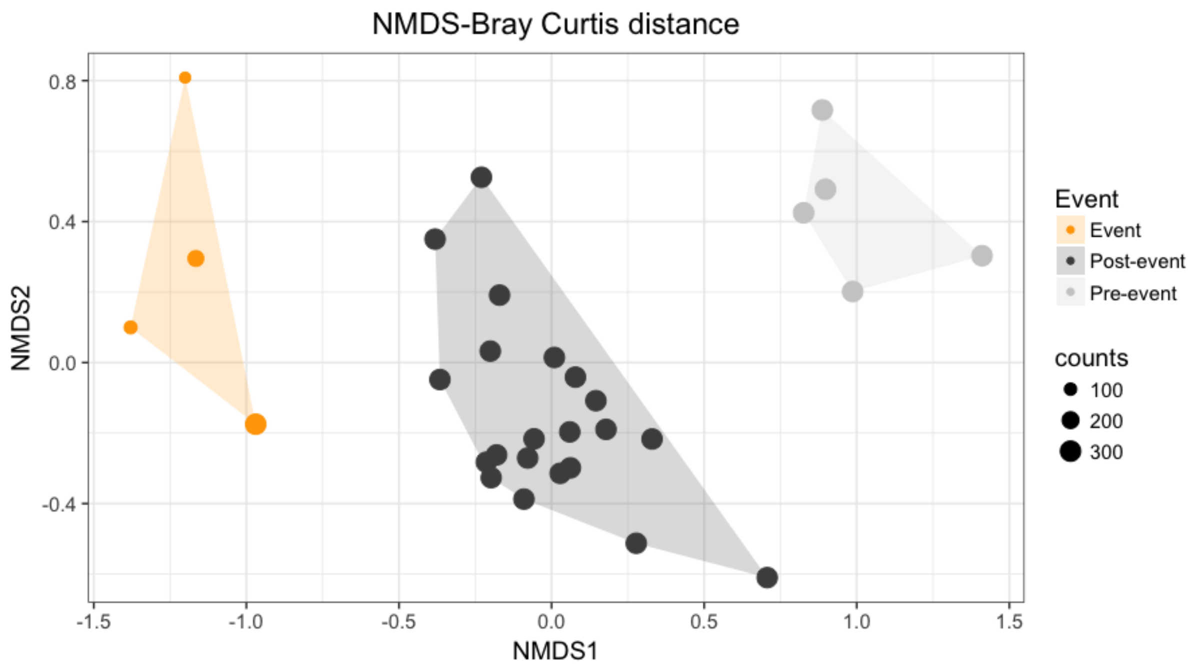

Fig. S5. Non-metric multidimensional scaling (NMDS) analysis of diatom assemblages showing the clear differences between diatoms within the rapid deposition event (orange symbols) and those deposited in pre- (grey symbols) and post- (black symbols) event sediments under conditions of normal lacustrine sedimentation.

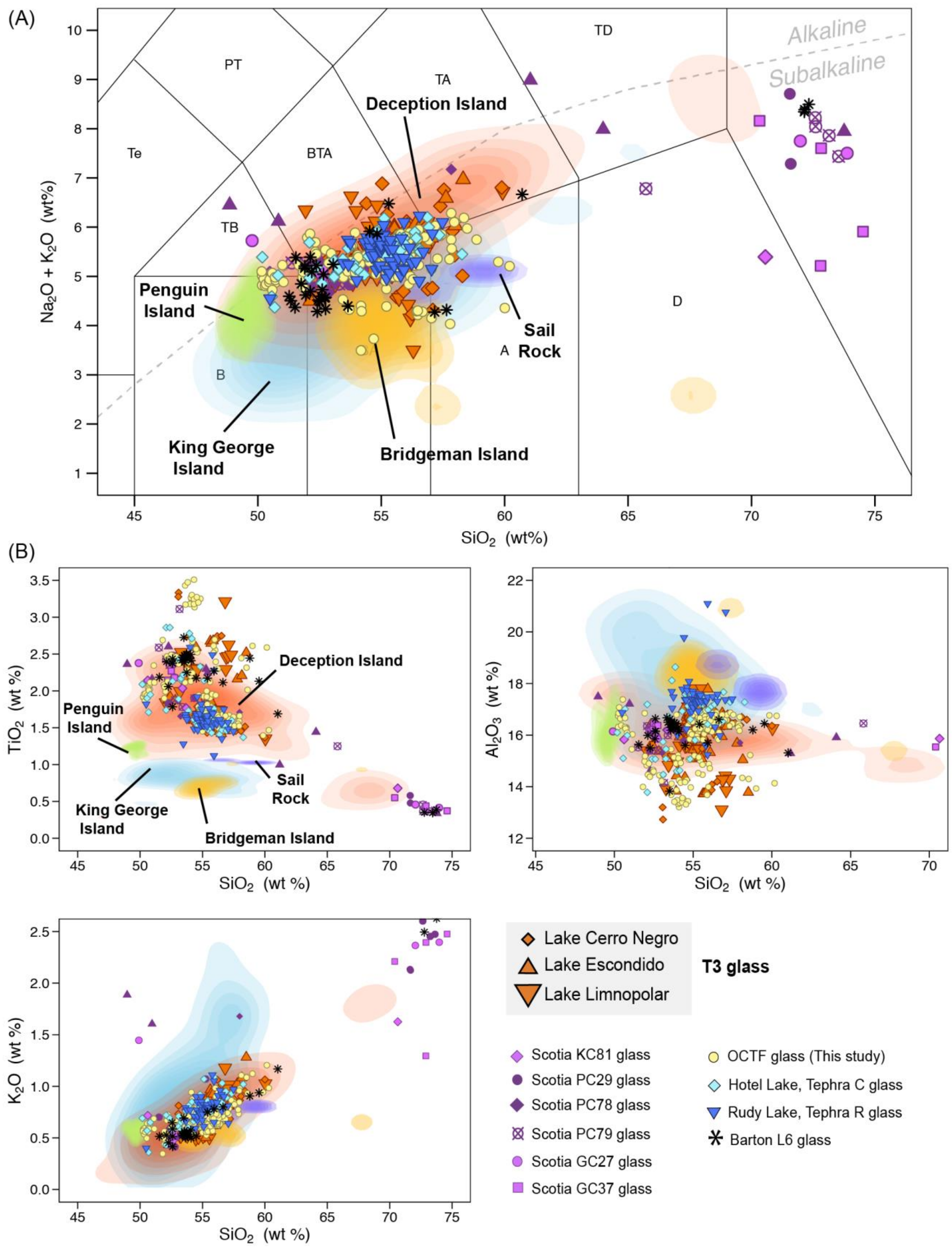
(A)
Te
PT
TA
TD
BTA
TB
B
A
D
Alkaline
Subalkaline
Deception Island
Penguin Island
Sail Rock
King George Island
Bridgeman Island
$Na_2O + K_2O$ (wt%)
SiO_2 (wt%)
(B)
TiO_2 (wt %)
Al_2O_3 (wt %)
K_2O (wt %)
SiO_2 (wt %)
Lake Cerro Negro
Lake Escondido
Lake Limnopolar
T3 glass
Scotia KC81 glass
Scotia PC29 glass
Scotia PC78 glass
Scotia PC79 glass
Scotia GC27 glass
Scotia GC37 glass
OCTF glass (This study)
Hotel Lake, Tephra C glass
Rudy Lake, Tephra R glass
Barton L6 glass

Fig. S6. Correlating the glass compositions for caldera collapse event tephra from Deception Island and sediment cores from published studies around the Antarctic Peninsula. The shaded areas show compositional fields (bulk rock) of nearby volcanic centres, including Penguin Island (green), King George Island (blue), Deception Island (red), Bridgeman Island (yellow) and Sail Rock (lilac). T3 samples from Byers Peninsula lakes Escondido, Cerro Negro and Limnopolar (this study); Barton L6: Barton Peninsula, King George Island (unpubl. data); Hotel Lake, Fildes Peninsula and Rudy Lake, Potter Peninsula , King George Island[43]; Scotia GC-027, GC-037, PC-029, PC-078, PC-079, KC-081: Scotia Sea[16, 21]. (A) Total Alkali vs. Silica diagram (TAS) (ref. 53). Major elements normalized to 100% (anhydrous) with Fe distributed between FeO and Fe_2O_3 following ref. 54. Grey dashed line discriminates between the alkaline-subalkaline fields[55]. (B) Major elements vs. SiO_2 content Harker Diagrams. Major element compositions have been normalised to 100% in anhydrous base with Fe as FeOt. See Supplementary file 2 for details on composition and latitude-longitude coordinates of the rock samples. This figure was generated with RStudio Version 1.0.143 (https://www.rstudio.com/) using ggplot2 package Version 2.1.9000[77]. Final layout of this figure was achieved using Adobe Illustrator CC 2015.3.1 (Copyright © 1987–2016 Adobe Systems Incorporated and its licensors).

Table S1.
List of radiocarbon ages constraining the chronology of Deception Island tephra in Byers Peninsula lakes. Data from lakes Escondido, Chester and Cerro Negro are from ref. 30 and those from Lake Limnopolar are from ref. 31. Strat. depth: stratigraphic depth, the depth in the composite core sequence of a given sample. Cal a BP: the median probability calibrated age, in years before present, where the present equals 1950 A.D.

Lake Escondido (n = 17)

**Lab Code**	**Core**	**Strat. depth (midpoint)**	**Material**	**Radiocarbon age**	**Cal a BP**	**2σ err. (±)**
ULA-4657	N/A	Surface	Moss	modern	n/a	n/a
ULA-4650	02-01g	10.9	Moss	1485 ± 20	1330	30
ULA-4651	03–02	11.1	Moss	1605 ± 15	1460	25
ULA-4256	02-01g	16.2	Moss	1830 ± 20	1720	60
ULA-4704	03–02	16.8	Moss	1810 ± 20	1660	60
ULA-4248	03–02	22.5	Moss	2010 ± 15	1920	30
ULA-4702	03–02	26.8	Moss	2195 ± 20	2150	50
ULA-4654	03–02	33.0	Moss	2375 ± 20	2350	30
ULA-4655	03–02	37.8	Moss	2595 ± 20	2710	30
ULA-4247	03–02	43.6	Moss	2600 ± 20	2720	30
ULA-4653	03–02	48.3	Moss	2925 ± 20	3010	70
ULA-4652	03–02	54.3	Moss	3220 ± 20	3400	50
ULA-4663	03–02	58.3	Moss	3325 ± 25	3510	70
ULA-4255	03–02	64.8	Moss	3630 ± 15	3890	50
ULA-4254	03–02	131.6	Moss	3765 ± 15	4060	50
ULA-4703	03–02	138.2	Moss	4265 ± 20	4750	30
ULA-4243	03–02	143.1	Moss	4600 ± 20	5160	50

Lake Chester (n = 7)

**Lab Code**	**Core**	**Depth (midpoint)**	**Material**	**Radiocarbon age**	**Cal a BP**	**2σ err. (±)**
ULA-4656	n/a	Surface	Moss	Modern	**n/a**	n/a
ULA-4251	CH12_0501g	51.4	Moss	3665 ± 20	**3930**	25
ULA-4249	CH12_0501g	30.05	Moss	1140 ± 20	**1000**	60
ULA-4242	CH12_0501g	22.1	Moss	1125 ± 15	**970**	60
ULA-4252	CH12_0401	20.1	Moss	3670 ± 15	**3940**	30
ULA-4244	CH12_0801	5	Moss	3685 ± 20	**3960**	50
ULA-4253	CH12_0801	125	Moss	4575 ± 20	**5160**	30

Lake Cerro Negro (n = 5)

**Lab Code**	**Core**	**Strat. Depth (midpoint)**	**Material**	**Radiocarbon age**	**Cal a BP**	**2σ err. (±)**
ULA-4658	n/a	Surface	Moss	Modern	**n/a**	n/a
ULA-4246	CN12_0301g	4.5	Moss	1855 ± 15	**1740**	20
ULA-4701	CN12_0301g	16.9	Moss	1955 ± 20	**1860**	50
ULA-4250	CN12_0301g	31.1	Moss	2690 ± 15	**2760**	30
ULA-4245	CN12_0301g	41.6	Moss	3010 ± 20	**3130**	50

Table S1. ***continued***

Lake Limnopolar (n = 25)

**Lab Code**	**Core**	**Combined depth**	**Material**	**Radiocarbon age**	**Cal a BP**	**2σ err. (±)**
Poz-39062	08D01	13.3–13.5	Moss	555 ± 30	**530**	25
Poz-39063	08D02	19.5–19.7	Moss	565 ± 30	**535**	30
Poz-39064	08D03	26.2–26.7	Moss	580 ± 30	**540**	25
Poz-39066	08D04	33.2–33.4	Moss	850 ± 30	**720**	50
Poz-39067	08D05	39.0–39.7	Moss	1045 ± 35	**910**	35
Poz-39068	08D06	48.7–49.3	Moss	1280 ± 30	**1150**	50
Poz-39070	08D07	56.4–57.0	Moss	1445 ± 30	**1310**	40
Poz-39071	08D08	65.0–65.5	Moss	1665 ± 35	**1520**	90
Poz-39072	08D09	75.8–76.4	Moss	1990 ± 30	**1900**	50
Poz-39073	08D10	85.0–85.3	Moss	2150 ± 35	**2090**	80
Poz-39074	08D11	94.3–94.8	Moss	2475 ± 30	**2480**	70
Poz-39075	08D12	97.9–98.3	Moss	2915 ± 35	**3000**	100
Poz-39076	08D13	102.3–102.5	Moss	2790 ± 35	**2840**	80
Poz-39077	08E01	104.9–105.4	Moss	2815 ± 35	**2870**	90
Poz-39255	08D14	100.0–110.5	Moss	3210 ± 35	**3390**	70
Poz-39080	08E02	112.3–112.8	Moss	3440 ± 35	**3640**	80
Poz-39081	08E03	122.1–122.5	Moss	3625 ± 35	**3890**	80
Poz-39082	08E04	130.0–130.4	Moss	4150 ± 35	**4640**	90
Poz-39083	08E05	140.3–140.7	Moss	4770 ± 40	**5470**	60
Poz-39084	08E07	154.0–154.4	Bulk sed.	10470 ± 70	**12280**	190
Poz-39085	08E09	180.5–181.1	Bulk sed.	9100 ± 70	**10220**	140
Poz-39086	08E10	188.3–188.5	Moss	4670 ± 40	**5400**	90
Poz-39087	08E11	190.7–191.2	Moss	4680 ± 40	**5400**	90
Poz-39088	08E12	200.2–200.6	Moss	5610 ± 40	**6360**	70
Poz-39090	08E13	205.2–205.4	Moss	6700 ± 50	**7530**	90

Table S2.
Indicator species analysis results for the three defined periods: pre-collapse event (*Pre-event*), rapid post-seismic sedimentation event (*Event*), and *post-event* sediments. The number "1" in each column represents the period(s) for which a given taxon is considered to be an indicator species ($p \leq 0.05$). The r.g-index indicates the value of the r-equalised index for each species. Aerophilic and soil species typical of the rapid post-seismic deposition event are in bold.

**Taxon**	**Pre-event**	**Event**	**Post-event**	**r.g-index**	***p*-value**
Achnanthes exigua	**1**	0	0	0.658	0.005
Diatomella balfouriana	**1**	0	0	0.637	0.015
Pinnularia gemella	**1**	0	0	0.503	0.045
Psammothidium abundans	**1**	0	0	0.958	0.005
Chamaepinnularia gerlachei	0	**1**	0	0.776	0.005
Craticula petradeblockiana	0	**1**	0	0.587	0.020
Luticola australomutica	0	**1**	0	0.564	0.035
Mayamaea* cf. *atomus* var. *permitis	0	**1**	0	0.553	0.030
Nitzschia paleacea	0	**1**	0	0.771	0.005
Nitzschia perminuta (complex)	0	**1**	0	0.923	0.005
Fragilaria capucina (complex)	0	**1**	**1**	0.803	0.005
Navicula australoshetlandica	0	0	**1**	0.569	0.025
Microcostatus naumanii	0	0	**1**	0.634	0.025
Pinnularia australomicrostauron	0	0	**1**	0.570	0.025
Brachysira minor	0	0	**1**	0.619	0.015
Diadesmis inconspicua	0	0	**1**	0.752	0.005
Psammothidium germainii	0	0	**1**	0.669	0.030
Psammothidium papilio	0	0	**1**	0.792	0.005
Psammothidium subatomoides	0	0	**1**	0.544	0.045
Planothidium renei	0	0	**1**	0.626	0.035
Sellaphora seminulum	0	0	**1**	0.675	0.010
Staurosira cf. *alpestris*	0	0	**1**	0.638	0.010
Staurosirella pinnata	0	0	**1**	0.695	0.005
Halamphora oligotraphenta	**1**	0	**1**	0.664	0.010
Achnanthidium minutissimum	**1**	0	**1**	0.655	0.020
Navicula cremeri	**1**	0	**1**	0.509	0.035
Psammothidium incognitum	**1**	0	**1**	0.601	0.045

Table S3. Sites with tephra and/or rapid sedimentation events that were correlated to the Deception Island caldera collapse by their chronostratigraphy (Chron.) and/or geochemistry (Geochem.). The first column indicates the code number used in Fig. 1 of the main manuscript. Age uncertainties are reported as 2σ ranges for recalibrated radiocarbon ages. ECM: Electrical conductivity measurement.

Fig. 1 Code	Age (cal a BP)	2σ range	Correlation type	Location	Feature	Core type	Depth	Features/ Comments	Reference
1	3995	200	Chron.	Epica Dronning Maud Land	ECM peak - source unattributed	Ice	296.29 m	Original age recalibrated to AICC 2012 time scale[36]	38, 39
2	3969	100	Chron.	Plateau Remote	Sulphate peak - source unattributed	Ice	195.74 m	Six sulphate peaks between 3953 and 4090 BP	41
3	3942	600	Chron.	Vostok – BH8	ECM peak - source unattributed	Ice	111.39 m	Original published age recalibrated to AICC 2012 time scale[36]	40
4	4004	200	Chron.	Epica Dome C	ECM peak - source unattributed	Ice	144.93 m	Original published age recalibrated to AICC 2012 time scale[36]	37
5	3998	130	Chron.	Talos Dome - TALDICE	ECM peak - source unattributed	Ice	317.32 m	Original published age recalibrated to AICC 2012 time scale[36]	35
6	3910	200	Chron.	James Ross Island	Deception Island tephra	Ice	344.47 m	Two other tephra at 3800 and 4100 BP	18
7	3870	220	Chron. & Geochem.	Bransfield Basin	Deception Island tephra	Marine (PC-61)	201 – 211 cm		57
8	3730	70	Chron. & Geochem.	W. Bransfield Basin	Tephra WBB 2, 201–211 cm.	Marine (JPC32)	365 cm		58
9	Between 3370 and 10670 BP (uncalibrated)		Geochem.	Scotia Sea	Deception Island tephra	Marine (PC-078)	35 cm		16, 21

10	Between 3370 and 10670 BP (uncalibrated)		Geochem.	Scotia Sea	Deception Island tephra	Marine (PC-079)	180 cm		16, 21
11	Between 3370 and 10670 BP (uncalibrated)		Geochem.	Scotia Sea	Deception Island tephra	Marine (KC-081)	21 cm		16, 21
12	Between 3370 and 10670 BP (uncalibrated)		Geochem.	Scotia Sea	Deception Island tephra	Marine (PC-029)	78 cm		16, 21
13	Between 3370 and 10670 BP (uncalibrated)		Geochem.	Scotia Sea	Deception Island tephra	Marine (GC-027)	31 cm		16, 21
14	3950	130	Chron. & Geochem.	Elephant Island	Tephra	Moss bank	116 cm		59
15	"base c. 4 kyr BP" (uncalibrated)	n/a	Chron. & Geochem.	Hotel Lake, Fildes Penin., King George Island	Deception Island tephra (60 cm thick) and rapid sedimentation event	Lake	85 – 240 cm	Interruption of lacustrine sediment-ation by 1.55 m gravity flow, absence of diatoms	42, 43
16	"between 3800 & 5200 yr BP" (uncalibrated)	n/a	Chron. & Geochem.	Rudy Lake, Potter Penin., King George Island	Deception Island tephra and rapid sedimentation event	Lake	73 – 120 cm	Interruption of lacustrine sediment-ation by 0.45 m gravity flow	42, 43
17	Bounding ages: 4320 and 3310	190 / 220	Chron. & Geochem	Tiefersee, Fildes Penin., King George Island	Period of apparent rapid sedimentation (100 cm deposition)	Lake	55 – 157 cm	Allochthonous sediment deposition; terrestrial diatoms; shifts in TOC content	44, 60
18	Not determined		Geochem.	Lake L6, Barton Penin., King George Island	Tephra B, Deception Island	Lake	113 – 130 cm		Unpubl. data
19	3875	130	Chron.	Annenkov Island, South Georgia	Rapid sedimentation event (215 cm thick)	Lake	256 – 471 cm	Average of 13 radiocarbon ages	61

ISOTOPIC EVIDENCE FOR T3 AS THE CALDERA FORMING ERUPTION

Oxygen and deuterium ($\delta^{18}O$, δD) stable isotope analyses provided essential information about the potential influence (isotopic equilibria) of non-magmatic fluids on the primary magma composition. Results show (i) that ^{18}O fractionation among the three samples is negligible – typical of such high-T magmas; and (ii) a progressively increasing influence from T3 to T1 of non-magmatic waters (i.e., oceanic) as revealed by the shift to heavier D values. Given the significant difference in D content between magmatic rock and oceanic water, any contact between the two is immediately registered (in terms of chemical disequilibrium) in the magmatic composition (*cf.* refs 24, 62, 63). Tephra T3, for which the δD falls within the range of magmatic waters (-51.2‰; Fig. S4), does not show any evidence of contact with non-magmatic waters. By contrast, tephra T2 δD (i.e., -26.3‰) suggests a limited amount of contact with non-magmatic waters (heavier values than T3) as indicated by its position within the range for active volcanoes; T1 (i.e., -4.4‰,) has much heavier D values than T2 and T3 (toward SMOW values) due to a significant influence of non-magmatic (oceanic) water. These results provide supporting evidence for a rapid eruptive event that allowed ^{18}O equilibrium in the magma system along ascent, but a rapid D disequilibrium because of the arrival of oceanic waters into the magmatic system as a function of different eruptive-collapse episodes in the history of the island. Given the huge difference in D/H content between a magmatic rock and oceanic water, any contact between the two waters is suddenly registered (in terms of chemical disequilibrium) in the magmatic fingerprint (see papers in refs. 24, 62 and 63). $\delta^{18}O$ and δD results reveal that (i) ^{18}O fractionation among the three samples is negligible –typical at such high T–, and (ii) sample T3, within the magmatic water stability field, shows no evidence of contact with any extra magmatic waters. Sample T2 received a certain amount of extra-magmatic waters (heavier values than T3)

falling within the active volcanoes stability field; and T1 gives D values much heavier than T2 and T3 (toward the SMOW values) due to a significant influence of extra magmatic –meteoric– waters.

LAKE LIMNOPOLAR RADIOCARBON AGES AND RESERVOIR EFFECT

The sediments of Lake Limnopolar contained the same stratigraphic sequence of tephra as well as a pronounced, rapid massive sedimentation event superposed on tephra T3 (Fig. 3, ref. 31). However, ^{14}C dates for correlated tephra were significantly older in Lake Limnopolar than those of the other lakes. The potential for old-carbon effects in certain Antarctic Peninsula lakes due to the contribution of glaciers, snowbanks, permafrost and catchment materials has long been identified as a potential problem[14]. Several catchment characteristics of Lake Limnopolar, distinct from the other study lakes, make it susceptible to the influence of such ^{14}C-depleted ancient carbon, including a pronounced division in both the catchment size and catchment-lake area ratio of Lake Limnopolar relative to all other lakes. Lake Limnopolar's catchment area is between 6.4 and 39 times larger than those of lakes Chester, Escondido and Cerro Negro, while these latter three lakes have catchment-lake ratios between 2.4 and 5.0, compared with a ratio of 26.2 for Lake Limnopolar. Put differently, while the other three lake surfaces represent between 20.0 and 41.1% of their total catchment area, Lake Limnopolar represents only 3.8%. Lake Limnopolar thus covers a much smaller portion of a much larger catchment, making it far more susceptible to the effects of allochthonous carbon deposition. Moreover, Lake Limnopolar's catchment includes a large area of deltaic origin; these environments are known to store ancient organic matter[64]. Lakes Escondido and Chester were therefore used to constrain the age of the

caldera collapse event, given that they consistently returned ages tightly clustered between 3900 and 4000 cal a BP.

EVENT BEDS AND DIATOM ANALYSIS OF PALAEOSEISMIC ACTIVITY

Evidence for the terrestrial origin of event bed material

We analysed several depositional proxies in order to determine the genesis of the rapid sedimentation event. Biological, geochemical and radiochronological data showed significant differences within the event bed sediments, each suggesting that sediments in the event beds were of terrestrial origin. The most likely mechanism for this rapid deposition of terrestrial sediment is mass wasting. All evidence is consistent with the transport of catchment material to the lake following mobilisation by a seiche resulting from a major seismic event: the large magnitude earthquakes that accompanied the caldera collapse eruption.

Diatom evidence of catchment mass-wasting

Identifications of Antarctic diatom species were based on taxonomic literature developed specifically from the South Shetland Islands and the Antarctic Peninsula region and conform to the emerging notion of a diverse flora rich in endemic species[65-70]. Non-metric multidimensional scaling (NMDS) was used to represent the ordering relationships among samples in a small number of dimensions (with similar samples plotting close to one another and dissimilar samples far apart) rather than the exact distance among samples[71]. Some samples were marked by low abundances or an absence of diatom frustules; only samples which reached a minimum threshold of 70 identified diatom valves were used for statistical analyses. Diatom community data was standardised (percentages) to calculate the Bray-Curtis dissimilarly matrix, which was used to

run NMDS using the Vegan package (version 2.3-1) in R (version 3.2.2). Characteristic diatom species were identified for each period (Pre-Event, Event and Post-event) using the ecological indicator value[72]. We allowed all possible combinations of periods and selected the combination for which the species could best be used as an indicator. Species could therefore be indicative of more than one period (group). We used the r-equalised index, as it avoids the potential problem of unbalanced sampling and can detect both negative and positive preferences of species for each period, or combinations of periods[72]. Indicator species analysis was performed using the Indicspecies package (version 1.7.5) in R.

There were three distinct periods in Lake Limnopolar sediments: prior to (*Pre-Event*; Fig. S6), during (*Event*) and following (*Post-Event*) the rapid sedimentation event. Diatom assemblages in *Pre-* and *Post-Event* sediments were dominated by taxa that are typical of large lakes on the Byers Peninsula plateau, as expected with normal lacustrine deposition[26], including *Achnanthes exigua* Grunow, *Achnanthidium minutissimum* (Kützing) Czarnecki, *Diatomella balfouriana* Greville, *Fragilaria capucina* Desmazières, *Psammothidium abundans* (Manguin) Bukhtiyarova & Round, *P. papilio* (Kellogg et al.) Kopalová & Van de Vijver, *P. subatomoides* (Hustedt) Bukhtiyarova & Round and *Sellaphora seminulum* (Grunow) Mann. Interestingly, *Post-Event* assemblages (Fig. S6) differed from *Event* diatoms but also from *Pre-Event* assemblages, which may indicate an ecological reorganisation due to the perturbation of algal communities by the eruption and its associated post-seismic event.

In contrast to abundant deposition in *pre-* and *post-Event* sediments, diatoms were scarce in sediments laid down during the rapid deposition event; the minimum threshold of identified

valves for inclusion statistical analyses was not reached in all samples. In all cases, however, event samples were characterised by strikingly different assemblages than those in lacustrine sediments (Fig. S6) and were dominated by species observed in soils, mosses and seepage samples in the Antarctic and sub-Antarctic regions (Table S2), including *Chamaepinnularia gerlachei* Van de Vijver & Sterken, *Craticula petradeblockiana* Van de Vijver et al., *Luticola australomutica* Van de Vijver, *Mayamaea* cf. *atomus* var. *permitis* Kützing, *Nitzschia paleacea*, and the *Nitzschia perminuta* complex (Table S2; refs 27, 65, 73-76).

The abrupt shifts between lacustrine and aerophilic/terrestrial diatoms indicate major inputs from catchment sources during the rapid deposition event. Given that the assemblage changes occur abruptly (i.e., there are no intermediate assemblages or gradual replacements) it is implausible that they resulted from decreases in lake level due to climatic factors. Allochthonous deposition is thus the only remaining explanation for the rapid replacement of lacustrine diatoms by terrestrial taxa in the sediment record.

EVIDENCE FOR THE WIDESPREAD FOOTPRINT OF THE DECEPTION ISLAND CALDERA COLLAPSE ERUPTION

In order to uncover the scope of the effects of the Deception Island caldera collapse eruption, we examined the scientific literature for significant events at and about 3980 cal a BP. We searched for evidence of tephra and geochemical events in ice and sediment cores from around Antarctica, as well as of sedimentological changes indicating rapid deposition in lake sediment cores in the South Shetland Islands and beyond. Geochemical compositions were used, where available, to corroborate tephra correlations, however these data were not available in all cases (See Table

S3). Where published ages were originally presented uncalibrated, if sufficient information (i.e. error ranges) was available we calibrated lake sediment and terrestrial samples using the SHCal04 curve for the Southern Hemisphere[45]. We did not attempt to calibrate marine ^{14}C ages where no local reservoir correction was suggested by the original authors. All ages presented in Table S3 are calibrated unless otherwise noted.

We found 18 separate records from around Antarctica in the literature that we correlated to the Deception Island caldera collapse. Our comparison of the major element geochemistry of tephra with OCTF and Byers Peninsula T3 samples (Fig. S6) confirms the link of Deception Island caldera collapse eruptive materials with marine and lacustrine tephra from around the region. Six ice cores contained glaciochemical volcanic signals or tephra synchronous with the collapse event, including James Ross Island (tephra, 3910 ± 200 y BP; ref. 18) and Plateau Remote (sulphate peak, 3969 ± 100 y BP; ref. 41), as well as an electrical conductivity peak correlated between the EPICA Dronning Maud Land, EPICA Dome C, Talos Dome TALDICE and Vostok cores[35, 37-40], covering an area that reached 4660 km from Deception Island, with ages (converted to the AICC2012 chronology; ref. 36) of 3995 ± 200 y BP, 4004 ± 200 y BP, 3998 ± 130 y BP and 3942 ± 600 y BP, respectively (Fig. 1, Table S3). The ice core ages were extremely well matched with our inferred age for the Deception Island caldera collapse, but no major element geochemistry was available to further substantiate these correlations.

Seven marine sediment cores across a >1350 km transect of the Bransfield Basin and Scotia Sea also recorded the Deception Island caldera collapse eruption (Fig. 1, Table S3). Major element geochemistry indicated that each of these tephras resulted from eruptions of Deception Island;

five were thought to have been produced during the caldera collapse eruption[16, 21]. However, the challenges inherent in dating Antarctic marine sediments, including variable local reservoir corrections and uncertainty about core-top recoveries, prevented the development of age constraints for this tephra beyond broad age ranges (Table S3). Two tephra in the Bransfield Basin that were geochemically matched to Deception Island, and with greater age control, were dated at 3870 ± 220 cal a BP and 3730 ± 70 cal a BP based on foraminiferal ^{14}C and palaeomagnetic ages, respectively[57, 58].

There is abundant evidence in the South Shetland Islands for the widespread effects of the caldera collapse event. In terrestrial environments, Björck *et al*.[59] recorded a distinct tephra from a Deception Island eruption that was deposited in a moss bank on Elephant Island 3950 ± 130 cal a BP. Several lakes around King George Island also contain Deception Island tephra, rapid sedimentation events, or both[42-44, 60]. These sites, located downwind according to the westerlies that dominate in the region, preserve Deception Island tephra reaching 60 cm thickness as well as superposed gravity flow deposits that in some lakes reached 1.6 m (Table S3). The bounding ages for these stratigraphic features are in agreement with our inferred age for the Deception Island caldera collapse, although their chronologies were often developed based on uncalibrated ages from conventional radiocarbon analysis, with few ^{14}C dates to constrain the stratigraphies. Moreover, in some studies no error values are presented, precluding our calibrating the ages. Finally, further afield on Annenkov Island in South Georgia, Strother *et al*. (ref. 61) recorded 215 cm of sediment deposition with coeval radiocarbon ages throughout at 3875± 130 cal a BP. This site is over 1650 km distant from Deception Island, and no tephra was identified in association with this sudden deposition to prove its relation to the caldera collapse. However,

although we cannot currently suggest a mechanism for the coincidence, given the position of Annenkov Island along the margin of the Scotia Plate the synchronicity of this rapid deposition event with the Deception Island caldera collapse is intriguing.

Supplementary references

56. Taylor, H. P. Oxygen isotope studies of hydrothermal mineral deposits in *Geochemistry of hydrothermal ore deposits* (ed. Barnes, H. L.) 109–142 (Holt, Rinehart and Winston, 1967).

57. Heroy, D. C., Sjunneskog, C. & Anderson, J. B. Holocene climate change in the Bransfield Basin, Antarctic Peninsula: evidence from sediment and diatom analysis. *Antarct. Sci.* **20,** 69–87 (2008).

58. Willmott, V., Domack, E. W., Canals, M. & Brachfeld, S. A high resolution relative paleointensity record from the Gerlache-Boyd paleo-ice stream region, northern Antarctic Peninsula. *Quaternary Res.* **66,** 1–11 (2006).

59. Björck, S. *et al.* Stratigraphic and paleoclimatic studies of a 5500-year-old moss bank on Elephant Island, Antarctica. *Arct. Alp. Res.* **23,** 361–374 (1991).

60. Matthies, D., Mäusbacher, R. & Storzer, D. Deception Island tephra: a stratigraphical marker for limnic and marine sediments in Bransfield Strait area, Antarctica. *Zentralblatt für Geologie und Paläontologie* **1,** 153–165 (1990).

61. Strother, S. L., *et al.* Changes in Holocene climate and the intensity of Southern Hemisphere Westerly Winds based on a high-resolution palynological record from sub-Antarctic South Georgia. *Holocene* **25,** 263–279 (2015).

62. Valley, J. W., Taylor, H. P. & O'Neil, J.R. (eds.) Stable isotopes in high temperature geological processes. *Rev. Geochem. Mineral.* **16 (**1986).

63. Bindenman, I. Oxygen isotopes in mantle and crustal magmas as revealed by single crystal analysis. *Rev. Mineral. Geochem.* **69,** 445–478 (2008).

64. Goni, M. A., Yunker, M. B., Macdonald, R. W. & Eglinton, T. I. The supply and preservation of ancient and modern components of organic carbon in the Canadian Beaufort Shelf of the Arctic Ocean. *Mar. Chem.* **93,** 53–73 (2005).

65. Van de Vijver, B. & Mataloni, G. New and interesting species in the genus *Luticola* D.G. Mann (Bacillariophyta) from Deception Island (South Shetland Islands). *Phycologia* **47,** 451–467 (2008).

66. Zidarova, R., Van de Vijver, B., Quesada, A. & de Haan, M. Revision of the genus *Hantzschia* (Bacillariophyceae) on Livingston Island (South Shetland Islands, Southern Atlantic Ocean). *Plant Ecol. Evol.* **143,** 318–333 (2010).

67. Kopalová, K., Elster, J., Nedbalová, L. & Van De Vijver, B. Three new terrestrial diatom species from seepage areas on James Ross Island (Antarctic Peninsula region). *Diatom Res.* **24,** 113–122 (2009).

68. Van de Vijver, B. & Zidarova, R. Five new taxa in the genus *Pinnularia* sectio Distantes (Bacillariophyta) from Livingston Island (South Shetland Islands). *Phytotaxa* **24,** 39–50 (2011).

69. Van de Vijver, B. *et al.* Revision of the genus *Navicula* s.s. (Bacillariophyceae) in inland waters of the Sub-Antarctic and Antarctic with the description of five new species. *Phycologia* **50,** 281–297 (2011).

70. Sterken, M. *et al.* An illustrated and annotated checklist of freshwater diatoms (Bacillariophyta) from Livingston, Signy and Beak Island (Maritime Antarctic Region). *Plant Ecol. Evol.* **148,** 431–455 (2015).

71. Legendre, P. & Legendre, L. *Numerical Ecology*. Amsterdam: Elsevier (1998).

72. De Cáceres, M., Legendre, P. & Moretti, M. Improving indicator species analysis by combining groups of sites. *Oikos* **119,** 1674-1684 (2010).

73. Van de Vijver, B. & Beyens, L. Freshwater diatoms from Ile de la Possession (Crozet Archipelago, sub-Antarctica): an ecological assessment. *Polar Biol.* **22,** 178–188 (1999).

74. Van de Vijver, B., Zidarova, R. & de Haan, M. Four new *Luticola* taxa (Bacillariophyta) from the South Shetland Islands and James Ross Island (Maritime Antarctic Region). *Nova Hedwigia* **92,** 137–158 (2011).

75. Hamsher, S., Kopalova, K., Kociolek, J.P., Zidarova, R. & Van De Vijver, B. The genus *Nitzschia* on the South Shetland Islands and James Ross Island. *Fottea* **16,** 79–102 (2016).

76. Van de Vijver, B., Frenot, Y. & Beyens, L. Freshwater diatoms from Île de la Possesion (Crozet Archipelago, Subantarctica). *Bibliotheca Diatomologica* **46,** 1–412 (2002).

77. Wickham, H. ggplot2: Elegant Graphics for Data Analysis. Springer-Verlag New York, (2016).
